# Supplementary material for: Evaluating the safety and patient impacts of an artificial intelligence command centre in acute hospital care: a mixed-methods protocol
Source: BMJ Open. 2022 Mar 1;12(3):e054090. doi: 10.1136/bmjopen-2021-054090 (PMC8889317; doi:10.1136/bmjopen-2021-054090)
Supplement: Supplementary data [file bmjopen-2021-054090supp001.pdf]

Appendix 1-Research Aim-Activities Matrix

Below is a matrix that indicates which research activities address which research aims and objectives

				Patient-safety time-series analysis	Patient-flow process mining	Data quality assessment	Unstructured observation	Structured observation	Stakeholder interview	Cross-industry consultation	CIO survey	Mixed-method synthesis	Dissemination
**Aim 1**	Evaluate the impact of the CC on patient safety, hospital operational efficiency and related organisational processes.	**a**	Describe (qualitatively) and evaluate (statistically) any effect on patient safety, including monitoring of deteriorating patients and sub-optimal care pathways, risk of harm due to cancellation/delays and situational awareness in safety-critical areas such as the emergency department.	X			X	X	X				
		**b**	Describe (qualitatively) and evaluate (statistically) any effect on patient flow, including capacity-demand ratio, transfer delays, bed utilisation, timely discharge and cancellations of scheduled care.		X		X	X	X				
		**c**	Qualitatively investigate any effect on organisational processes, such as situational awareness, operational decision-making, risk and coordination/communication across organisational units, from multiple stakeholder perspectives.				X	X	X				
**Aim 2**	Understand the process of implementation and integration of the CC and associated data infrastructure and organisational processes within the primary study site	**a**	Using qualitative methods, describe the process of development and implementation of the CC, including critical implementation factors and any unintended consequences.				X	X	X				
		**b**	Through ethnographic methods, investigate the process by which the CC system and outputs are embedded at all levels of the organisation, from frontline operations to strategic quality and safety governance.					X					
		**c**	Develop and validate a logic model for this health informatics intervention that maps system preconditions, processes, technology and outcomes, at the primary study site.									X	
		**d**	Describe (statistically and qualitatively) the effect of the CC implementation on the local data environment, including data infrastructure, quality and integration (i.e. system interoperability).			X	X	X	X				
**Aim 3**	Elicit cross-sector and cross-industry perspectives on hospital command and control technologies to contextualise the findings from the	**a**	Review and understand command and control processes in non-healthcare safety-critical operations and the key principles and contextual factors that may influence transferability of these models into a hospital setting.							X			

	primary study site for broader application.	**b**	Survey the perceptions of senior health informatics professionals on current command and control processes, viability of novel "mission-control" systems, data readiness and potential implementation barriers.								X		
**Aim 4**	Synthesise the research findings into practical outputs that will engage service stakeholders and inform future investment and practice	**a**	Share learning concerning cross-industry and empirical findings on the costs-benefit of investment within NHS management and Chief Information Officer networks.										X
		**b**	Construct an empirically-informed implementation framework that describes contextual factors and implementation pathway for development of centralised, data-driven mission-control systems in acute care, including data infrastructure maturity.									X	
