# Supplementary material for: Evaluating the safety and patient impacts of an artificial intelligence command centre in acute hospital care: a mixed-methods protocol
Source: BMJ Open. 2022 Mar 1;12(3):e054090. doi: 10.1136/bmjopen-2021-054090 (PMC8889317; doi:10.1136/bmjopen-2021-054090)
Supplement: Supplementary data [file bmjopen-2021-054090supp002.pdf]

Appendix 2- Study Steering Group membership

Below are the details of the members of the Study Steering Committee for the NIHR HS&DR project entitled "Evaluating the safety and patient impacts of an AI Command Centre in the NHS" (NIHR129483).

Name	Affiliation	Reason for nomination
Iain Buchan (Chair)	University of Liverpool; University of Manchester (Honorary)	Executive Dean, Institute of Population Health Sciences; Director of Digital Strategy and Partnerships, Liverpool Health Partners; Chair of Public Health and Clinical Informatics
Paul Charnley	Wirral NHS Teaching Hospitals Trust	Director of IT and Information at Wirral NHS Teaching Hospitals Trust
Sarah Culkin	NHS England; NHSX	Artificial Intelligence lead for NHS England
Cindy Fedell	Regional Chief Information Officer at Northwestern Ontario Hospitals	Former Chief Information Officer at Bradford Teaching Hospitals NHS Foundation Trust
Hamish Fraser	Brown University, USA	A health informatics academic and clinical lead for OpenMRS, the most-widely used open-source medical record system
Charles Koo	Envive Technology, Ltd.; Visiting Scholar (Stanford University); Visiting Expert (National Taiwan University)	Implemented large scale Artificial-Intelligence, hospital systems at six hospitals in Shanghai, China.
Farah Magrabi	Macquarie University	Associate Professor Australian Institute of Health Innovation
Sue Mason	Sheffield University; Sheffield NHS Teaching Hospitals Trust	Professor of Emergency Medicine at Sheffield University and clinician at Sheffield NHS Teaching Hospitals Trust
Mark Sujan	University of Warwick; Human Factors Everywhere	Human Factors expert
Hilary Thompson	NIHR Yorkshire and Humber Patient Safety Translational Research Centre	Patient representative and NIHR YH PSTRC Lay Leader
Sean White	NHS Digital	Senior Safety Engineer
Patrick Waterson	Loughborough University	Reader in Human Factors and Complex Systems
