# Supplementary material for: Evaluating the safety and patient impacts of an artificial intelligence command centre in acute hospital care: a mixed-methods protocol
Source: BMJ Open. 2022 Mar 1;12(3):e054090. doi: 10.1136/bmjopen-2021-054090 (PMC8889317; doi:10.1136/bmjopen-2021-054090)
Supplement: Supplementary data [file bmjopen-2021-054090supp003.pdf]

Research Data Leeds http://researchdata.leeds.ac.uk/ email: researchdataenquiries@leeds.ac.uk
Oct 2019

University of Leeds Data Management Plan (DMP) Template

Researcher Name	Owen Johnson
Project Title	Evaluating the safety and patient impact of an Artificial Intelligence Command Centre in the UK National Health Service
Faculty	Engineering and Physical Sciences
KRISTAL Reference Number (if applicable)	118684
Supervisor(s) name (if applicable)	n/a
Funder	National Institute for Health Research
Scheme	
Research Start Date	1st March 2021
Research End Date	30th Sept 2022
Ethical review number	Not available, yet
DMP review due	3rd May 2021

Date	Version	Author	Change notes
11th Nov 2020	V1.0	Ciarán McInerney	n/a
16th Dec 2020	V2.0	Ciarán McInerney and Carolyn McCrorie	Added data management plan for qualitative work.
10th Feb 2021	V3.0	Ciarán McInerney and Carolyn McCrorie	Added details of the standards with which Yorkshire and Humber Care Record's Google Cloud Platform are compliant.

Please provide a brief overview of your project including proposed research methods

AI Command Centre in Bradford NHS Bradford Teaching Hospitals NHS Foundation Trust is implementing an artificial intelligence (AI) command centre in Bradford Royal Infirmary, which is regarded as the first of its kind in Europe. The command centre follows an approach successfully used in the USA (Johns Hopkins, Baltimore) and Canada (Humber River, Toronto) to provide real-time, rapid response to clinical, management and patient-flow challenges. Similar to an air traffic control command centre, hospital staff work together in a purpose-built operations room and monitor a 'wall of analytics' of high-definition screens that display real-time data from the hospital's clinical systems. The team review, monitor and react to the 'big picture' of how efficiently patients are flowing through the hospital, where bottlenecks might occur, where pressure is building and where safety breaches are predicted. The command centre software makes use of AI technologies that are refined through operation to provide increasingly more intelligent alerts and warnings. There is a very limited evidence base for this form of digital technology in hospitals but an increasing belief that AI can and should play a key role in transforming and modernising the NHS. Our research team in the NIHR Yorkshire & Humber Patient Safety Translational Research Centre (YH-PSTRC) are based in Bradford and in a unique position to collect and study the evidence. Research Framework We propose a mixed-method study with three phases over 18 months, commencing in 2020. In the first phase, we will carry out initial scoping work with relevant stakeholders. The second and third phases will involve longitudinal case studies describing the impact of an AI command centre on safety. We will also

Adapted from University of Cambridge DataTrain project & University of Edinburgh MANTRA project

conduct a mixed-method comparison with hospitals such as Calderdale and Huddersfield NHS foundation Trust, which has shared systems and learning through close collaboration with Bradford. Outputs Our aim in this study is to provide a robust academic evaluation of the AI Command Centre so that other hospitals and healthcare providers can consider how best to exploit this emerging technology. The findings from our study will be reported to the NHS trusts, including local dissemination of findings. They will also be reported in a white paper for distribution to NHS Chief Information Officers, in academic publications, to NIHR as part of the annual report for funding body for the YH-PSTRC and as funders for this specific project and dissemination of findings via the NIHR PSTRCs' networks.

1. What data will be produced? What data will be used from other sources?

With respect to the quantitative component of the project:

1. Study on patient safety.
Collect:

- **What?** - Routinely-collected electronic health record data that informs the measures of patient safety identified during early qualitative work. This data is created and stored in Bradford Teaching Hospital NHS Trust's electronic health record system, Cerner Millennium. This data will be de-identified and aggregated within the Yorkshire Health and Care Record, from which we will request extracts.
- **Why?** - This data is created by the hospital system being studied and so reflects the safety performance of the hospital system.

Create:

- **What?** - Summary statistics of the distribution and dynamics of patient-safety measures at both sites. Visualisations of the values. Models of variable dynamics. We will also create R scripts, which are text documents containing instructions to run commands in the R statistical programme.
- **Why?** - The measures of patient-safety are the variables of interest in this study. The R scripts are needed to process the data. The visualisations will facilitate investigations and some will be used to communicate the research.

2. Study on patient flow.
Collect:

- **What?** - Routinely-collected electronic health record data created and stored in Bradford Teaching Hospital NHS Trust's electronic health record system, Cerner Millennium. This data will be de-identified within the Yorkshire Health and Care Record, from which we will request extracts.
- **Why?** - Pseudonymised patient identifier, a description of a clinical event, and the timestamp for the event are the minimal requirements to build a process model of a patient flow through a hospital.

Create:

Adapted from University of Cambridge DataTrain project & University of Edinburgh MANTRA project

CC BY NC SA

- **What?** - We will create R scripts, which are text documents containing instructions to run commands in the R statistical programme. These R scripts will produce R files containing process models and summary statistics describing the process models. Visualisations of the models and values.
- **Why?** - The models describe patient flow, which is a marker of patient safety. These models need to be evaluated so we compute summary statistics of their performance. The R scripts are needed to process the data. The visualisations will facilitate investigations and some will be used to communicate the research.

3. Study on data quality.
Collect:

- **What?** - Routinely-collected electronic health record data that are presented or used to inform variables that are presented on the AI Command Centre tiles. This data is created and stored in Bradford Teaching Hospital NHS Trust's electronic health record system, Cerner Millennium. Identifying the variables of interest will require an audit of the tiles of interest, which will be conducted in early qualitative work.
- **Why?** - This data is created by the hospital system being studied. We wish to assess how the quality of this data might have changed as the Command Centre was gradually implemented.

Create:

- **What?** - We will be using the Weiskopf et al. (2017) tool for assessing the quality of the data, which will create the data indicated in the table below, as per their 3x3 data-quality matrix. Each cell of the matrix will inform a visualisation of the data. We will also create R scripts, which are text documents containing instructions to run commands in the R statistical programme.

	Complete	*Correct*	*Current*
Patients	Counts and percentages of Variables, Times with recorded data, and Overall points of data present.	Details of expected value limits for the variables across patients. A citation for the source of the expectation. Counts and percentages of patients whose data are within the value limits.	Details of the expected period within which the data were recorded. A citation for the source of the expectation. For each non-static variable, Counts and percentages of patients whose data do not fall within the desired date range. Summary statistics of the discrepancies between actual recording dates and desired date ranges (mean, median, mode, 1st quartile, 3rd quartile, interquartile range, minimum,

Adapted from University of Cambridge DataTrain project & University of Edinburgh MANTRA project

			maximum, standard deviation, variance)
Variables	Counts and percentages of Patients with the variable, Times with the variable present, and Overall points of data present.	Details of criteria that indicate concordance for each variable. A citation for the source of the expectation. Counts and percentages of patients who meet each criterion. Counts and percentages of patients whose data violate any of the criteria.	Details of the expected sequence or intervals between events. A citation for the source of the expectation. Counts and percentages of patients that meet expectations, for each variable. Counts and percentages of patients that meet all expectations.
Times	Counts and percentages of Patients with data recorded for that time, Variables with data recorded for that time, and Overall points of data present.	Details of criteria that indicate valid changes over time, for each variable. A citation for the source of the expectation. Counts and percentages of patients who meet each criterion. Counts and percentages of patients whose data violate any of the criteria.	The I-score for each variable for which regularity is to be assessed. Summary statistics for the I-score across all variables (mean, median, mode, 1st quartile, 3rd quartile, interquartile range, minimum, maximum, standard deviation, variance). The IxN-score to measure "effective data points".

- **Why?** - The data created using the Weiskopf et al. (2017) tool for assessing the quality are the variables of interest in this study. The R scripts are needed to process the data. The visualisations will facilitate investigations and some will be used to communicate the research.

With respect to the qualitative component of the project:

1. Case description and unstructured observation

Collect:

- **What?** -Ethnographic observations within the Command Centre (CC) Unit. Up to 36 hours over 4-hour periods.
- **Why?** -In order to immerse and sensitise the research team to the context of hospital operational command and control. The data will help us to understand events and actions as they unfold from the actor's perspective (and the meanings that CC users attach to them).

Create:

- **What?** -Researcher field notes

Adapted from University of Cambridge DataTrain project & University of Edinburgh MANTRA project

- **Why?** -To draw upon the concepts of Grounded Theory in which we will adopt an inductive qualitative analysis approach to understand way in which the CC system integrates within the broader hospital information and operational planning systems in a formal model grounded in our data.

2. System-wide structured observation

Collect:

- **What?** -Ethnographic observations within and beyond the Command Centre. Up to 10 hours observation each of specific tracer issues/professional roles that represent interaction with CC processes and outputs.
- **Why?** -In order to explore the impact of the CC beyond the operations room and at all levels of the organisation, including micro-level (frontline clinical workflow in specific specialties), meso-level operational planning (e.g. bed management) and macro-level strategic planning (e.g. use of data in quality and safety governance).

Create:

- **What?** -Researcher field notes
- **Why?** -To draw upon concepts of Realist Evaluation in our analysis to understand usability of the system in context.

3. Longitudinal stakeholder and process evaluation

Collect:

- **What?** -Qualitative research interviews with up to 24 staff relative to the initiative
- **Why?** -To evaluate the efficacy of the system from multiple user perspectives

Create:

- **What?** -Interview transcripts
- **Why?** -To draw upon a process evaluation framework approach to analysis in order to understand intervention mechanisms, implementation processes, interaction with context and overall outcomes.

4. Cross-industry study

Collect:

- **What?** -Qualitative research interviews with up to 10 consultants in safety critical industries
- **Why?** -To elicit and apply knowledge from high-risk industry to the development of strategies for implementing command and control centres to improve quality and safety

Create:

- **What?** -Interview transcripts

Adapted from University of Cambridge DataTrain project & University of Edinburgh MANTRA project

- **Why?** -To draw upon a process evaluation framework approach to analysis in order to understand intervention mechanisms, implementation processes, interaction with context and overall outcomes.

5. Survey study

Collect:

- **What?** -Survey of a sample of Chief Information Officers in acute care across England and Wales
- **Why?** -To capture views on current practices in data-supported operational planning

Create:

- **What?** -Survey responses captured through UoL Online Surveys
- **Why?** -The data is required to understand variations in electronic data-facilitated command and control beyond the 2 research sites

2. Where will data be stored? How will data be structured? Include file formats and approximate volume.

Where will data be stored?
The electronic health records used in the quantitative work will not be copied for storage or back-up, by the research team. Instead, it will be hosted by the Yorkshire and Humber Care Record and accessed via their virtual research environment. The Yorkshire and Humber Care Record system is built on Google Cloud technology with Identity Access Management following the principle of least privilege, i.e. minimum permissions of access and functionality. Google Cloud technology is compliant with GDPR, ISO/IEC 27001, ISO/IEC 27017, ISO/IEC 27018, ISO/IEC 27701, NHS Digital Commercial Third-Party Information Governance Requirements, UK's Cloud Security Principles. Further details are available at https://cloud.google.com/security/compliance.
Toward the end of the project, summative research output for publications and all R scripts used for data processing will be exported from the Yorkshire and Humber Care Record portal and stored on the University of Leeds SAN (Storage Area Network), which comprises enterprise level disk storage and file servers located in physically secure data centres with appropriate fire suppression equipment. Snapshots are taken every day at 10pm (and accessible for 1 month). A second level of snapshots is taken every month and are kept for 11 months. Snapshots are user recoverable from the desktop.
A full back-up to tape is taken once every month and an incremental copy to backup tape is taken every night (and kept for 28 days). Every quarter, the most recent set of full dump tapes are moved to a long-term storage facility where they are kept for 12 months. Tapes are initially stored in on-campus fireproof safes and then moved to off-campus secure locations. The SAN is located behind the University's Institutional firewall to protect against external attacks.
During the life of the qualitative work, the data will be stored on the University of Leeds SAN. The audio-recording equipment will be encrypted. Survey data will be stored in UoL Online Surveys
After the project has completed, data will be offered to the University of Leeds Research Data Repository (Research Data Leeds) or another appropriate data repository service in order to ensure the data can be shared, reused and cited beyond the end of the project. Research Data Leeds holds deposited data for a minimum of 10 years and datasets are associated with digital object identifiers (DOIs).

How will data be structured?

Adapted from University of Cambridge DataTrain project & University of Edinburgh MANTRA project

Research Data Leeds http://researchdata.leeds.ac.uk/ email: researchdataenquiries@leeds.ac.uk
Oct 2019

With respect to the 'collected' quantitative data (in so far as 'data collection' refers to the method by which data is obtained):

- **Format** - The data is expected to be accessible in the Yorkshire Health and Care Record portal as a CSV file or in one of the SQL database file types, which we would subsequently transform into a CSV file type.
- **Volume** - The size of the dataset will be informed mostly by the count of patients, rather than by the count of variables. Both hospital sites under study typically see in the range of 70,000 - 80,000 unique patients every month, each with at least two events (admission and discharge). This translates to a volume of data in the order of millions of observations across multiple variables. File sizes are likely to be in the order of MBs.

With respect to the created quantitative data:

- **Format** - The data will be stored as R files and CSV files, outputted using RStudio. The R format will facilitate analysis while the CSV formats are preferred to transferability to other software. All visualisations will be stored in PNG and JPEG formats. The JPEG formats are have smaller file sizes and will be used only when the higher quality PNG format is not supported.
- **Volume** - Likely no more than low double figures of megabytes.

With respect to the collected qualitative data:

- **Format** – The interview transcripts and field notes will be stored as files within NVivo 12 version. Survey data will be stored in SPSS software.
- **Volume** – The size of the data set will be informed by the number of interviews undertaken, size of the field notes and number of responses to the survey.

With respect to the created qualitative data:

- **Format** - The interview transcripts and field notes will be stored as files within NVivo 12 version. Survey data will be stored in SPSS software.
- **Volume** – The size of the data set will be informed by the number of interviews undertaken, size of the field notes and number of responses to the survey.

3. Access to data during the project. Give details of collaborators and any controls.

During the life of the quantitative work, access to the 'collected' data will be controlled by the Yorkshire and Humber Care Record, with whom our research team will have a contract stipulating the terms of use of the Yorkshire and Humber Care Record portal. The created data that will occasionally be exported and stored on University of Leeds' Storage Area Network. These data will only be accessible by university staff with user privileges and password access.
During the life of the qualitative work, the data will be stored on UOL SAN. Only members of the research team will have access via user privileges and password access.
After the project has completed, the data that will be stored with the data repository service will only be accessible on request and following approval criteria that will be co-developed by the research team and the data repository service.

4. Ethics and legal compliance: are there any 'special' requirements for your data? Any contractual or consent issues? Key policies (internal and external)

Adapted from University of Cambridge DataTrain project & University of Edinburgh MANTRA project

Research Data Leeds http://researchdata.leeds.ac.uk/ email: researchdataenquiries@leeds.ac.uk
Oct 2019

Consent – all interviewees will give informed consent for the interview to be audio-recorded and transcribed.
5. How will data be documented and described? Methodologies and protocols.<br>All folders will contain a README in a TXT file format that explains what files are in the folder. Each file will be named, its provenance (including source and steps taken to process it), and details of any restrictions on sharing. No formal standard will be adhered to.
6. Training and support<br><br>All researchers have completed UoL training in Information Security Essential.
7. What are the plans for data sharing beyond project partners? Include justification if some of your data needs to be restricted. Include data and code. Include repository.<br><br>After the project has completed, the data that will be stored with a data repository service will only be accessible on request and following approval criteria that will be co-developed by the research team and the data repository service. Data will be made available via the University of Leeds data repository and, where possible, as supplementary material accompanying academic publications.<br>The electronic health records used in the quantitative work will not be made available outside of the Yorkshire and Humber Care Record in which it was accessed.
8. What Intellectual Property will be generated? How will IP be protected and exploited?<br>We are not expecting to generate Intellectual Property beyond the academic outputs produce vai the research process.
9. Who is responsible for managing the data? What resources will you need?<br><br>The Principal Investigator will have ultimate responsibility for data management during the project but the quantitative and qualitative study leads will have day-to-day responsibilities. After the project, the data will be managed by the University of Leeds data repository staff.
10. Ongoing data curation / data housekeeping - you may find it useful to include a retention table<br><br>All summative data used to communicate research findings in published output will be stored in the University of Leeds data repository. This repository holds deposited data for a minimum of 10 years and datasets are associated with digital object identifiers (DOIs).

End of Project

At the end of a project and/or before you leave the institution, you should ensure that data and research materials are deposited with the School or a trusted data repository and documented in such a way that they can be found and understood.

Dataset name	Location	Person responsible

Adapted from University of Cambridge DataTrain project & University of Edinburgh MANTRA project

BY NC SA

Research Data Leeds http://researchdata.leeds.ac.uk/ email: researchdataenquiries@leeds.ac.uk
Oct 2019

University of Leeds Data Management Plan (DMP) Template: Prompt Sheet

1. What data will be produced or used? (Including original software)

- ☐ What physical data will you study? (e.g. artefacts, samples, paper archives, etc.)
- ☐ What digital data will you generate? (e.g. field-notes, images, spreadsheets, audio interviews, survey data, annotated bibliography, etc.)
- ☐ What original software will you generate?
- ☐ What third party data will you reuse?

2. Where will data be stored? How will data be structured?

- ☐ Estimate how much data you will produce over time – do you have enough storage?
- ☐ Do you know what University storage is available and how to access it?
- ☐ What file formats and software will you use?
- ☐ Do you have a logical file naming convention and directory structure?
- ☐ How will you use versioning so you can identify the current version of documents / data?
- ☐ How will data generated in the field be saved to safe University storage?

3. Access to data during the project. Give details of collaborators and any controls.

- ☐ Have you discussed data sharing with your research collaborators/ supervisor?
- ☐ Who needs to access data during the research? How will they access data?
- ☐ Do you need a data sharing agreement? (see also section 4.)

4. Ethics and legal compliance: are there any 'special' requirements for your data?

- ☐ Have you read the University's Information Protection Policy? Data must be assessed for sensitivity and storage in line with this policy https://it.leeds.ac.uk/it?id=kb_article&sysparm_article=KB0011140
- ☐ Are you familiar with the University's advice on data protection and GDPR? https://dataprotection.leeds.ac.uk/
- ☐ Does your research funder have specific data management and sharing requirements?
- ☐ Are there other policies and protocols you need to be aware of and observe? For example, NHS codes of practice?
- ☐ Will you anonymise your data?
- ☐ Should some data be destroyed? When and how?
- ☐ How and where will you record any participant consents and/or contractual requirements which impact data management and sharing? The DMP can be a good place to record this information.

5. How will data be documented and described? Methodologies and protocols.

- ☐ Will others understand your data? Write documentation. Make sure table and spreadsheet values are clearly labelled.
- ☐ What information about data collection methodology will be recorded?
- ☐ Is it important for the research to be reproducible? Why/why not? What additional documentation will be required?
- ☐ Will you write software? Where will this be documented and stored for future use?

Adapted from University of Cambridge DataTrain project & University of Edinburgh MANTRA project

cc BY NC SA

Research Data Leeds http://researchdata.leeds.ac.uk/ email: researchdataenquiries@leeds.ac.uk
Oct 2019

6. Training and support

- What training do you need for data gathering, organisation, analysis or presentation?
- Are there relevant courses available at the University? Online? Who can provide support?

7. What are the plans for data sharing beyond project partners?

- Have you considered reasons for and against sharing data? Will data be openly available to everyone or will there be access restrictions?
- If your research involves people, have you obtained appropriate consent for data sharing?
- Can your data be released immediately, or should you embargo (delay access to) the data?
- How long will / should data be available for?
- Will you use a data repository? Which one? Are there subject specific data repositories in your field?

8. What IPR will be generated? How will IPR be protected and exploited?

- Will you be applying for a patent? Will your research have commercial applications? Do you need to contact the Commercialisation team in the Research and Innovation Service?
- Have you read the University Intellectual Property Policy? http://ris.leeds.ac.uk/downloads/download/600/university_of_leeds_ipr_policy

9. Who is responsible for managing the data? What resources will you need?

- Who is responsible for data at different stages in its lifecycle?
- On projects with complex data management requirements, different types of role should be specified.
- How will best practice and guidance be shared across the project partners?
- Are sufficient resources (skills, people, storage, technology) available to deliver your plan?

10. Ongoing data curation / data housekeeping - you may find it useful to include a retention table

- What data will you keep? Who decides?
- Where will data be kept and for how long.
- Who needs to know what data exists on the network, where it is, how it should be managed and how long it should be retained?

Don't forget to review and update your data management plan regularly

But I don't have any data! *Anything can become research data if it is used for research purposes – data is not just numbers on a spreadsheet. Think creatively about the materials you are using and producing: what could be shared with other researchers who are interested in your work; what could be reused to produce new insights? Any evidence or material which underpins or sheds light on your findings, your academic publications, your thesis or your project can be considered research data.*

Adapted from University of Cambridge DataTrain project & University of Edinburgh MANTRA project

CC BY NC SA
