# Supplementary material for: Evaluating the safety and patient impacts of an artificial intelligence command centre in acute hospital care: a mixed-methods protocol
Source: BMJ Open. 2022 Mar 1;12(3):e054090. doi: 10.1136/bmjopen-2021-054090 (PMC8889317; doi:10.1136/bmjopen-2021-054090)
Supplement: Author's manuscript [file bmjopen-2021-054090.draft_revisions.pdf]

BMJ Open is committed to open peer review. As part of this commitment we make the peer review history of every article we publish publicly available.

When an article is published we post the peer reviewers' comments and the authors' responses online. We also post the versions of the paper that were used during peer review. These are the versions that the peer review comments apply to.

The versions of the paper that follow are the versions that were submitted during the peer review process. They are not the versions of record or the final published versions. They should not be cited or distributed as the published version of this manuscript.

BMJ Open is an open access journal and the full, final, typeset and author-corrected version of record of the manuscript is available on our site with no access controls, subscription charges or pay-per-view fees (http://bmjopen.bmj.com).

If you have any questions on BMJ Open's open peer review process please email info.bmjopen@bmj.com

Evaluating the safety and patient impacts of an Artificial Intelligence Command Centre in acute hospital care: A mixed-methods protocol

Journal:	*BMJ Open*
Manuscript ID	bmjopen-2021-054090
Article Type:	Protocol
Date Submitted by the Author:	02-Jun-2021

Complete List of Authors:	McInerney, Ciarán ; Bradford Royal Infirmary<br>McCrorie, Carolyn; University of Leeds Faculty of Social Sciences, School of Psychology<br>Benn, Jonathan; University of Leeds<br>Habli, Ibrahim; University of York, Department of Computer Science<br>Lawton, Tom; Bradford Royal Infirmary<br>Mebrahtu, Teumzghi; University of Leeds Faculty of Engineering and Physical Sciences, School of Computing<br>Randell, Rebecca; University of Bradford, Faculty of Health Studies<br>Sheikh, Naeem; Wolfson Centre for Applied Health Research<br>Johnson, Owen; University of Leeds School of Computing, Computing

Keywords:	Health informatics < BIOTECHNOLOGY & BIOINFORMATICS, Information management < BIOTECHNOLOGY & BIOINFORMATICS, Information technology < BIOTECHNOLOGY & BIOINFORMATICS, Telemedicine < BIOTECHNOLOGY & BIOINFORMATICS

SCHOLARONE™ Manuscripts

Title

Evaluating the safety and patient impacts of an Artificial Intelligence Command Centre in acute hospital care: A mixed-methods protocol

Names and institutions

Name: Ciarán D. McInerney [CORRESPONDING AUTHOR]
Affiliation: School of Computing, University of Leeds, UK; Wolfson Centre for Applied Health Research, Bradford Royal Infirmary, Bradford, UK
Email: c.mcinerney@leeds.ac.uk

Name: Carolyn McCrorie
Affiliation: School of Psychology, University of Leeds, UK; Wolfson Centre for Applied Health Research, Bradford Royal Infirmary, Bradford, UK

Name: Jonathan Benn
Affiliation: School of Psychology, University of Leeds, UK; Wolfson Centre for Applied Health Research, Bradford Royal Infirmary, Bradford, UK

Name: Ibrahim Habli
Affiliation: Department of Computer Science, University of York, Deramore Lane, York, UK

Name: Tom Lawton
Affiliation: Bradford Teaching Hospitals NHS Foundation Trust, Bradford Royal Infirmary, Duckworth Lane, Bradford, UK

Name: Teumzghi Mebrahtu
Affiliation: School of Computing, University of Leeds, Leeds, UK

Name: Rebecca Randell
Affiliation: Faculty of Health Studies, University of Bradford, UK; Wolfson Centre for Applied Health Research, UK

Name: Naeem Sheikh
Affiliation: NIHR Yorkshire and Humber Patient Safety Translational Research Centre, Wolfson Centre for Applied Health Research, Bradford Royal Infirmary, Bradford, UK

Name: Owen Johnson
Affiliation: School of Computing, University of Leeds, UK; Wolfson Centre for Applied Health Research, Bradford Royal Infirmary, Bradford, UK

Abstract

Introduction
This paper presents a mixed methods study protocol that will be used to evaluate a recent implementation of a real-time, centralised hospital command centre in the UK. The Command Centre represents a complex intervention within a complex adaptive system. It could support better operational decision-making and facilitate identification and mitigation of threats to patient safety.

For peer review only

There is, however, limited research on the impact of such complex health information technology on patient safety, reliability and operational efficiency of healthcare delivery and this study aims to help address that gap.

Methods and analysis

We will conduct a longitudinal mixed-method evaluation that will be informed by public-and-patient involvement and engagement. Interviews and ethnographic observations will inform iterations with quantitative analysis that will sensitise further qualitative work. Quantitative work will take an iterative approach to identify relevant outcome measures from both the literature and pragmatically from datasets of routinely-collected electronic health records.

Ethics and dissemination

This protocol has been approved by the University of Leeds Engineering and Physical Sciences Research Ethics Committee (#MEEC 20-016) and the NHS Health Research Authority (IRAS No.: 285933). Our results will be communicated through peer-reviewed publications in international journals and conferences. We will provide ongoing feedback as part of our engagement work with local trust stakeholders.

Registration

This study is registered with Research Registry (#researchregistry6611).

WORD COUNT: 3,873

Strengths and limitations of this study

- We will explore how an implementation of a real-time, centralised hospital command centre will affect patient flow, enhance situational awareness, support operational decision-making and facilitate identification and timely mitigation of threats to patient safety.
- We will conduct a longitudinal mixed-method evaluation informed by a multidisciplinary co-investigator team and public-and-patient involvement and engagement.
- Interviews and ethnographic observations will inform iterations for quantitative analysis that will sensitise further qualitative work.
- We will develop a logic model to describe the command centre as a complex, health-informatics intervention, and provide ongoing feedback as part of our engagement work with local trust stakeholders.
- We will interpret our findings in the context of changes to the structures and processes of healthcare systems in response to the COVID-19 pandemic.

Introduction

Fragmented healthcare delivery adversely affects patient safety and care.[1,2] Health information technology might help information flow across fragmented organisations[3,4] but investment and adoption has been limited, and is not a completely sufficient solution.[5] The result is a complex socio-technical organisation that challenges healthcare delivery.[6] In the face of dynamic risks and organisational complexity, high-reliability organisations, like nuclear power plants and air traffic control systems, nevertheless demonstrate how safety can be maintained.[7,8]

For peer review only

Such high-reliability organisations use centralised “mission control” structures to integrate intelligence in real time and facilitate safe and efficient operational decision making. Within acute care settings, a hospital “command centre” or “mission control” is a relatively novel concept but one that is now achievable given recent advances in electronic health records, data integration and investment from commercial developers. There is however limited research on the impact of such complex health information technology on patient safety, reliability and operational efficiency of healthcare delivery. It is also unknown whether such centralised structures are appropriate for coordinating a hospital given that a) healthcare provision is not bounded within the hospital, unlike energy provision within a nuclear power plant, b) the system under ‘control’ is comprised of people, unlike non-living materials in off-shore oil, and c) healthcare operates on a demand-pull dynamic, rather than the supply-push dynamic.

The sociotechnical requirements for effective command centres have been studied in transport and military situations,[9] and there have been attempts to use artificial intelligence (AI) principles within command centres since Project Cybersyn in Chile as early as 1971.[10] There is a limited evidence base for this form of digital technology in healthcare[11] – although some successes have been reported in the USA[12] – but there is an increasing belief, supported by the Government’s Life Sciences Strategy, that AI should play a key role in transforming and modernising the NHS in the UK.[13,14] Thus, there is a need to understand how hospitals manage their operations through advances in health information technology, and how this affects the quality and safety of patient care.

There is a mismatch between the factors required for successful implementation, end users and provider perspectives.[17] The disruptions to workflow are significant challenges for users, particularly in systems that have limited modularity and configurability.[17] There have been very few published attempts to evaluate effectiveness, except for use as a clinical decision support tool[18] and most of the evidence originates from North America.[19] Given the major differences in the social, political and economic foundations of their healthcare system, it is important to explore whether these issues are relevant to the UK context.[20]

Even less is known about the potential of electronic health records developments to improve patient outcomes. Patient journeys are poorly understood, with aggregate statistics obfuscating detail. What has not been achieved is real-time command and control using the data generated by routine systems, despite a rise in the number of state-of-the-art dashboards, flow and simulation models.

Bradford Teaching Hospitals NHS Foundation Trust (BTHFT) has implemented a hospital Command Centre in the United Kingdom (UK), working with commercial suppliers from the USA. The Bradford AI Command Centre aims to provide faster and safer care by reducing unnecessary waiting by anticipating and avoiding bottlenecks in care delivery before they cause problems. Such AI command centres have the potential to improve future patient flow and safety, and research to understand the health service delivery, safety and operational factors should be considered an area of major importance for hospitals.

We hypothesise that the implementation and integration of a real-time, centralised hospital command centre will improve patient flow, enhance situational awareness, support operational decision-making and facilitate identification and timely mitigation of threats to patient safety. Supported by funding from the National Institute for Health Research (NIHR), we have proposed a mixed-methods project that combines ethnographic observations, qualitative process evaluation and quasi-experimental methods to study the evolving sociotechnical nature of the systems and processes within hospitals. Our protocol is theoretically informed by contemporary safety science theory concerning system resilience,[21,22] human factors models of situational awareness[23,24] and command-and-control in high reliability organisations.[25–27]

For peer review only

Aim and objectives

Our aim is to evaluate the safety and patient impacts of the Bradford AI Command Centre. Our objectives are:

1. To evaluate the impact of the Command Centre on patient safety, hospital operational efficiency and related organisational processes ('Impact Evaluation');
2. To understand the process of implementation and integration of the Command Centre with data infrastructure and organisational processes ('Process Evaluation');
3. To contextualise the findings through cross-sector and cross-industry perspectives on hospital command and control technologies ('Cross-industry perspectives'); *and*
4. To synthesise findings into practical outputs to engage service stakeholders and inform future investment and practice ('Synthesis and dissemination').

Further breakdown of the objectives is shown in Appendix 1, indicating the contribution of the research activities described in the next section.

Methods and analysis

Design and data collection

System implementations such the Bradford AI Command Centre are complex interventions into complex adaptive systems that could provide improvements but might also result in emergent unforeseen consequences.[28,29] We will conduct a longitudinal mixed-method evaluation informed by the multidisciplinary co-investigator team and public-and-patient involvement and engagement. A mixed-method approach is well suited to study complexity interventions (e.g. [30]) and the complex adaptive systems to which they are applied. Mixed-method approaches have been used to study information flow and organisational networks,[31] integration of organisational interventions,[32,33] effectiveness of service models,[34] and how health information technology affects communication,[35] patient monitoring,[36–38] care provision,[39] and clinical decision making.[40]

Our study comprises five sub-studies across five work streams (figure 1). The five sub-studies are conducted by the qualitative and quantitative work streams (WS3 and WS4 in figure 1). These work streams mutually inform each other as part of an iterative synthesis of findings, rather than solely a summative synthesis. Qualitative and quantitative work streams will work in parallel, with qualitative interviews and ethnographic observations informing iterations for quantitative analysis that will sensitise further qualitative work. Quantitative work will take an iterative approach to identify relevant outcome measures from the literature and pragmatically from the dataset. These will be subsequently verified by the qualitative work.

The main research activity will be guided by the project's Study Steering Group (see WS1 in figure 1) and by patient-and-public involvement and engagement (PPIE; see WS2 in figure 1). Our Study Steering Group is an independent body ensuring the project is conducted to the rigorous standards set out in the Department of Health's Research Governance Framework for Health and Social Care and the Guidelines for Good Clinical Practice.[41] The Study Steering Group will also facilitate stakeholder engagement in research and dissemination, and will advise on policy communications (see Ethics and dissemination, below). Membership of our Study Steering Group includes clinical, technical, commercial and academic healthcare representatives from the UK, Canada, USA, China, Australia (Appendix 2).

For peer review only

< Figure 1 Schematic overview of the research project's components. BRI = Bradford Royal Infirmary, CIO = Chief Information Officer, HS&DR = National Institute for Health Research (NIHR) Health Service and Deliver Research funding programme, PPIE = Patient and Public Involvement and Engagement, WS = work stream.>

Sub-study 1: Data quality

This sub-study will contribute to objectives 1 ('Impact Evaluation') and 2 ('Process Evaluation') based on the hypothesis that the introduction of the Command Centre will affect the awareness of, recording of and processing of electronic healthcare data, which are inextricably related to data infrastructure, operational efficiency, and organisational processes. We will apply Weiskopf *et al.*'s 3x3 matrix to assess the quality of healthcare data.[42] This framework maps Patient, Variables and Time data items in terms of Completeness, Correctness and Currency (in other words, presence, accuracy, and timeliness). Further detail on how to implement the 3x3 matrix is available in Weiskopf *et al*.[43] Our initial identification of variables will be informed by our qualitative sub-studies, and additional clinical input will inform the expected attributes of the data, e.g. plausible ranges, regularity, expected completeness.

Sub-study 2: Patient flow

This sub-study will contribute to objectives 1 ('Impact Evaluation') and 2 ('Process Evaluation') based on the hypothesis that operational efficiency, organisational processes and patient safety are affected by the flow of patients through the hospital. To study patient flow, we will use process-mining methods[44] to describe patients' journeys through their hospital care.[45] We will construct process models to represent patients' log of clinical events (see examples in dentistry,[46] oncology,[47] sepsis,[48] and primary care[49]). We will evaluate these models by comparing their performance when constructed using various process-mining algorithms. The performance of the models will be measured by:[50]

1. *Replay fitness*, a measure of how many traces from the log can be reproduced in the process model, with penalties for skips and insertions; range 0 – 1.
2. *Precision*, a measure of how 'lean' the model is at representing traces from the log. Lower values indicate superfluous structure in the model; range 0 – 1.
3. *Generalisation*, a measure of generalisability as indicated by the redundancy of nodes in the model. The more redundant the nodes, the more variety of possible traces that can be represented; range 0 – 1.

Patient flow as defined by the best-performing process model will be described using the multi-level approach of Kurniati *et al.*,[51] which include activity, trace and model measures. As all sub-studies progress, other measures of patient flow might be suggested for study, e.g. patient-level measures.

Sub-study 3: Patient safety

This sub-study will contribute to objectives 1 ('Impact Evaluation') and 2 ('Process Evaluation') by directly evaluating the differences in patient-safety outcomes before and after the implementation of the Command Centre. The evaluation will use longitudinal data-analysis methods, e.g. including interrupted time-series analysis and latent growth modelling. We will model trends behaviour before, during and after the implementation of the Command Centre, with consideration for the onset of the COVID-19 pandemic. Unobserved confounders will be handled by including a control

For peer review only

site from the same geographical region that uses the same electronic health record system, but which does not use a Command Centre.

We will approach the analysis in a responsive manner, adding or removing interrupts in response to unfolding understanding of the Command Centre's implementation, based on our qualitative process evaluation. Candidate variables of interest will include the Patient Safety Indicators from the Agency for Healthcare and Research Quality, e.g. pressure ulcer rate, in-hospital fall with hip fracture rate, post-operative sepsis rate.[52] These will be supplemented by variables of interest informed by the qualitative sub-studies and early PPIE workshops.

We assume that patient safety is influenced by the flow of patients and the quality of information (as encoded in electronic health records). Therefore, we also intend to use the aforementioned sub-studies on data quality and patient flow to inform clinically meaningful outcomes logically related to patient safety. These outcomes will be subject to longitudinal analysis like the patient safety outcomes.

Sub-study 4: Ethnography and qualitative interview

This study will contribute to objectives 1 ('Impact Evaluation') and 2 ('Process Evaluation'). Ethnographic enquiry has been selected to facilitate deep understanding of the technology in its broader social and organisational context, including human experience, engagement and interaction.[53,54] We aim to achieve a comprehensive description of how the Command Centre is integrated and embedded within the broader sociotechnical hospital system through observation of enacted working practices, communication, decision-making, and operating culture. In this sense, we will not simply be relying upon the model for the system implementation as planned by programme leads, but will explore the differences between work as intended and work as done[21] and describe any unintended consequences and implementation barriers as they emerge. The ethnographic and qualitative interview sub-study is comprised of three main research activities, detailed below.

Unstructured observations

We will conduct unstructured observations in the Command Centre to gather information of staff interactions with data, and the ways in which this influences decision-making. Through research field notes and interviews, we will seek to understand the role of the Command Centre in coordinating care, from the perspective of staff in and around the Command Centre. Two researchers will undertake up to 36 hours of observation, completed in 4-hour windows that represent different times of day and days of the week. Observation periods will be pre-specified through arrangement with Command Centre leads. In addition, the researchers will record incidents of observer effects to allow analysis of whether participants' awareness of the researchers' presence changed over time.[55] We will also explore behaviour and meaning around specific events, drawing upon the Critical Incident Technique.[56]

We will review emerging hospital policies and guidance related to the Command Centre (e.g. meeting minutes and operational procedures) where practicable as an alternative to data collection involving staff. A sampling framework to guide collection of documents will be informed through earlier qualitative interviews with Command Centre leads and iterated during the research process. Documents that meet inclusion criteria will be recorded in a document inventory and a data extraction template will be created to obtain the necessary information from the documents. The extracted data will be analysed through an inductive process to capture key developments in design and functioning of the Command Centre.

For peer review only

Longitudinal stakeholder and process evaluation

Our process evaluation framework[57] will systematically explore the experiences, beliefs, and expectations from multiple user perspectives in relation to operational planning and delineate the trajectories by which patient safety, operational and other intermediary outcomes are impacted by Command Centre processes.

We will use qualitative research interviews at multiple time points within the Command Centre programme. This will include building upon the ethnographic work to explore interactions with prior theory concerning how the Command Centre might works. The evaluation will address the factors that govern engagement with and use of this technology, using technology adoption theory.[58] The evaluation will also address the efficiency and effectiveness of processes for generating new intelligence for decision making and quality improvement, at the level of the hospital. A key output will be a logic model to describe the Command Centre as a complex, health-informatics intervention.

Sampling will be theoretically driven, based upon emerging insights from the structured observations, and will include Command Centre programme leads, key roles working in the centre, clinical leads in frontline areas interacting with the Command Centre, and organisational level stakeholders representing senior information systems, operational strategy, clinical governance, and financial interests. Up to 20 interviews will be undertaken at the study site focusing on two timepoints: one during the early phase of the project and the second towards the end of data collection. Representation of comparable roles will be sought at the control site, for comparative analysis of how the implicated functions are delivered in conventional operational planning processes.

Structured observations

We will undertake structured observations to inform use-cases of operational planning, control and decision-making in priority areas, at the hospital level. Our approach to structured observation will draw upon engineering use-case methodology[59] to understand usability of the system in context. We will follow key information from the Command Centre's visual displays to decision and actions taken by key professional roles. This will involve shadowing various roles, such as bed managers, risk management, quality assurance, clinical leads and others, as they act and make decisions based on Command Centre information. We will produce up to six use cases, based upon 60 hours of observation. We will utilise the use cases identified in structured observations as a probe to compare operational planning, control and decision-making in specific priority areas, with and without the support of a centralised Command Centre function, to enrich our understanding of how a Command Centre operates within a health service context.

Field notes and interview transcripts will be entered into NVivo (version 12.6) software to facilitate data management. Data analysis will comprise both inductive and deductive analyses, employing frameworks derived from prior theory and a comparative perspective across the two study sites.[60,61]

Relevant theoretical frameworks for sense making in our analysis will include models of situational awareness,[62] operational command and control, [63–65] sociotechnical evaluation, [66] High Reliability Organisations[7,64,67] and resilience in healthcare.[68,69] From the perspective of Situational Awareness theory, for example, we will seek to understand how the Command Centre enhances human perception of the environment and events within time and space, including projection of future states, and facilitates comprehension of meaning.[62]

For peer review only

Sub-study 5: Cross-industry consultation and CIO survey

The sub-study will contribute to objective 3 ('Cross-industry Perspectives'). We will undertake a cross-industry review as part of our work, comprising qualitative literature review and consultation with subject-matter experts in a range of safety-critical domains. Such an approach has been applied successfully in previous work, which sought to elicit and apply knowledge from high-risk industry to the development of incident reporting systems in healthcare.[70]

Data collection will involve scoping the literature in a range of domains for conceptual and empirical models of causal mechanisms for centralised command and control. We will additionally consult with up to 10 industry experts, including representatives of similar command centre programmes in other health systems, accessed through UK health care human factors and other professional networks. This process will be supported by the project's Study Steering Group. The results will be synthesised to inform analysis and interpretation of our data.

We will also conduct a survey of the perceptions of NHS information personnel in acute care across England and Wales to understand variations in electronic data-facilitated command and control within hospitals beyond the two research sites. The survey instrument will be informed by earlier qualitative research work and literature review. We will capture views on current practices in data-supported operational planning and control, the costs-benefits of investment in centralised command and control "centres", information/data readiness, implementation barriers and perceptions of the need for further development in this area.

The survey will be disseminated to NHS information personnel through the University of Leeds Online Surveys network. Data will be transferred into SPSS software to facilitate data analysis.

Patient and public involvement and engagement

Patient and public involvement and engagement is an integral part of our study design, delivery and dissemination. Figure 1 shows how the PPIE work stream is engaged throughout all phases of the project. Pre-study PPIE included a patient-and-public representative as co-applicant (NS), input on research design by the PPIE Research Fellow at the NIHR Yorkshire and Humber Patient Safety Translational Research Centre, and an informal survey of visitors to the hospital in which the Command Centre was implemented.

Early project PPIE activity includes workshops at the Command Centre and control site to engage PPIE representatives to give lay perspectives on care coordination in hospitals, to inform the development of interview questions for hospital staff, and to suggest measures of patient safety. Our PPIE co-applicant will support qualitative data analysis through review and further development of emerging themes in the dataset. Toward the end of the project, a joint workshop will host PPIE representatives from both hospital sites to help interpret findings and to draft a PPIE communication plan. Our PPIE Lay Leader and project co-applicant will also co-develop case descriptions with the qualitative research team and will co-author all publications to provide a PPIE perspective.

Sampling and recruitment

For the qualitative work, we will recruit relevant staff at the Command Centre and control site in key roles. NHS staff working in and around the Command Centre will be asked to take part in ethnographic observations. Up to 40 NHS staff will be interviewed, 20 from each site. We will sample ≤ 10 cross-industry experts for interviews, and ≤ 40 NHS hospital information personnel in England and Wales will be asked to take part in the survey. Sampling will be theoretically informed in

For peer review only

accordance with qualitative research practices to maximise variation in stakeholder perspectives. Potential participants will be identified through clinical leads and early observations.

For the quantitative work stream, we will use complete sampling of electronic health records within relevant periods. The duration of relevant periods will be informed by the initial case description and unstructured observations in the qualitative work, which will sensitise us to the information handled by the Command Centre.

Consent and data handling

Research participants will be told that they do not have to take part in the research if they wish and that they can withdraw up to the point that their data has been anonymised (< 2 weeks following research interviews; < 1 week following survey). The quantitative work will analyse routinely-collected healthcare records data. These data will have been de-identified and processed by the hospitals' data teams and accessed via Connected Yorkshire – an ethically approved regional integration of healthcare and other data available for research purposes.[71]

Limitations

This proposed protocol was planned prior to the COVID-19 pandemic, which has caused substantial changes to the structures and processes used in healthcare systems. Our sub-studies on data quality, patient flow and patient safety are intended to provide quantification of the influence of the Command Centre implementation but will be limited in their capability to distinguish such contributions from those motivated by the response to COVID-19. Our mixed-method approach and involvement from our international Study Steering Group will help to define the context of this turbulent period and to describe the processes of change in the hospitals studied. Under the epistemic constraints of our pre-COVID, funder-approved protocol, we will interpret our research through these contextual descriptions.

Ethics and dissemination

This protocol has been approved by the University of Leeds Engineering and Physical Sciences Research Ethics Committee (#MEEC 20-016) and the NHS Health Research Authority (IRAS No.: 285933). The protocol was developed by a multi-disciplinary team of co-applicants, including PPIE representatives, and by the NIHR as part of the NIHR 19/16 HSDR Digital Technologies to Improve Health and Care funding processes.

We will provide ongoing feedback as part of our engagement work with local trust stakeholders. At the end of the study, we will provide a report detailing design feedback to improve the implementation of the AI Command Centre at the Bradford site and more generally for the system supplier and the digital technology industry. Our results will be communicated through peer-reviewed publications in international journals and conferences.

To contribute to objective 4 ('Synthesis and Dissemination'), we will involve the research team, project co-applicants, the Study Steering Committee, stakeholders, and PPIE representatives consolidate research findings, PPIE perspectives and Study Steering Committee insight into outputs to appropriate audiences.

For peer review only

After the project has completed, data that is approved to do so will be offered to the University of Leeds Research Data Repository, in accordance with our Data Management Plan (Appendix 3).

Acknowledgements

This research is supported by the National Institute for Health Research (NIHR) Yorkshire and Humber Patient Safety Translational Research Centre (NIHR Yorkshire and Humber PSTRC). The views expressed in this article are those of the authors and not necessarily those of the NHS, the NIHR, or the Department of Health and Social Care.

Authors' contributions

JB, CMC, CMI and OJ conceptualised the study. All authors developed the research protocol, reviewed the initial funding bid, contributed to the response to funder queries, and reviewed this manuscript. CMI and CMC led the writing of this manuscript.

Funding statement

This project is funded by the National Institute for Health Research Health Service and Delivery Research Programme (NIHR129483).

Disclaimer

The views and opinions expressed therein are those of the authors and do not necessarily reflect those of the Health Service and Delivery Research Programme, NIHR, NHS or the Department of Health.

Competing interests

None declared.

References

1 Stange KC. The problem of fragmentation and the need for integrative solutions. *Ann Fam Med* 2009;**7**:100–3. doi:10.1370/afm.971

2 Castro-Sánchez E, Charani E, Drumright LN, *et al.* Fragmentation of care threatens patient safety in peripheral vascular catheter management in acute care- A qualitative study. *PLoS One* 2014;**9**. doi:10.1371/journal.pone.0086167

3 Nguyen L, Bellucci E, Nguyen LT. Electronic health records implementation: An evaluation of information system impact and contingency factors. *Int J Med Inform* 2014;**83**:779–96. doi:10.1016/j.ijmedinf.2014.06.011

4 Lau F, Bartle-Clar JA, Bliss G, *et al.* Improving usability, safety and patient outcomes with

For peer review only

health information technology: From research to practice. In: Lau F, Bartle-Clar JA, Bliss G, *et al.*, eds. *Information Technology and Communications in Health, ITCH 2019*. Victoria, Canada: : IOS Press 2019. https://pdfs.semanticscholar.org/8141/c5c3f62e1346cc200fbaf429d988b7130e04.pdf#page=521

5 Sujan MA, Scott P, Cresswell K. Digital health and patient safety: Technology is not a magic wand. *Health Informatics J* Published Online First: 2019. doi:10.1177/1460458219876183

6 Ramanujam R, Rousseau DM. The challenges are organizational not just clinical. *J Organ Behav* 2006;**27**:811–27. doi:10.1002/job.411

7 LaPorte T, Consolini P. Working in Practice but Not in Theory: Theoretical Challenges of "High-Reliability Organizations. *J Public Adm Res Theory* 1991;**1**:19–48.

8 Roberts K, Madsen P, Desai V, *et al.* A case of the birth and death of a high reliability healthcare organisation. *Qual Saf Heal Care* 2005;**14**:216.

9 Holwell S, Checkland P. An information system won the war. *IEE Proc Softw* 1998;**145**:95–9. doi:10.1049/ip-sen:19982194

10 Espejo R. Complexity and change: Reflections upon the cybernetic intervention in Chile, 1970-1973. *Syst Pract* 1990;**3**:303–13. doi:10.1007/BF01062734

11 Buch VH, Ahmed I, Maruthappu M. Artificial intelligence in medicine: current trends and future possibilities. *Br J Gen Pract* 2018;**68**:143–4. doi:10.3399/bjgp18X695213

12 Johns Hopkins Medicine. The Johns Hopkins Hospital Launches Capacity Command Center to Enhance Hospital Operations. 2016.https://www.hopkinsmedicine.org/news/media/releases/the_johns_hopkins_hospital_launches_capacity_command_center_to_enhance_hospital_operations (accessed 26 Jan 2021).

13 Bell, John. Life sciences: industrial strategy. Published Online First: 2017.https://assets.publishing.service.gov.uk/government/uploads/system/uploads/attachment_data/file/650447/LifeSciencesIndustrialStrategy_acc2.pdf

14 UK AI COUNCIL. AI Roadmap. 2021.https://www.gov.uk/government/publications/ai-roadmap

15 Biruk Senafekesh, Yilma Tesfahun, AndualemMulusew, *et al.* Health Professionals readiness to implement electronic medical record system at three hospitals in Ethiopia: a cross sectional study. *BMC Med Inform Decis Mak* 2014;**14**:1–8.

16 Gesulga JM, Berjame A, Moquiala KS, *et al.* Barriers to Electronic Health Record System Implementation and Information Systems Resources: A Structured Review. *Procedia Comput Sci* 2017;**124**:544–51. doi:10.1016/j.procs.2017.12.188

17 Sidek YH, Martins JT. Perceived critical success factors of electronic health record system implementation in a dental clinic context: An organisational management perspective. *Int J Med Inform* 2017;**107**:88–100. doi:10.1016/j.ijmedinf.2017.08.007

18 Black AD, Car J, Pagliari C, *et al.* The Impact of eHealth on the Quality and Safety of Health Care: A Systematic Overview. *PLoS Med* 2011;**8**:e1000387. doi:10.1371/journal.pmed.1000387

19 Johnson OA, Fraser HSF, Wyatt JC, *et al.* Electronic health records in the UK and USA. *Lancet* 2014;**384**:954. doi:10.1016/S0140-6736(14)61626-3

For peer review only

20 Protti D. Comparison of IT in general practice in 10 countries. *Heal Q* 2007;**10**:107–16.

21 Hollnagel E, Wears RL, Braithwaite J. From Safety-I to Safety-II: A White Paper. 2015.https://www.england.nhs.uk/signuptosafety/wp-content/uploads/sites/16/2015/10/safety-1-safety-2-whte-papr.pdf

22 Hollnagel E. *Safety-I and safety-II: the past and future of safety management*. Ashgate 2018.

23 Endsley MR. Measurement of situation awareness in dynamic systems. *Hum Factors* 1995;**37**:65–84. doi:10.1518/001872095779049499

24 Endsley MR. Situation awareness misconceptions and misunderstandings. *J Cogn Eng Decis Mak* 2015;**9**:4–32. doi:10.1177/1555343415572631

25 Weick KE, Sutcliffe K, Obstfeld D. Organizing for high reliability: Processes of collective mindfulness. In: Sutton R, Staw B, eds. *Research in Organizational Behavior*. JAI Press 1999. 81–123.

26 Cantu J, Tolk J, Fritts S, *et al.* High Reliability Organization (HRO) systematic literature review: Discovery of culture as a foundational hallmark. *J Contingencies Cris Manag* 2020;**28**:399–410. doi:10.1111/1468-5973.12293

27 Aboumatar HJ, Weaver SJ, Rees D, *et al.* Towards high-reliability organising in healthcare: A strategy for building organisational capacity. *BMJ Qual Saf* 2017;**26**:663–70. doi:10.1136/bmjqs-2016-006240

28 Sturmberg JP, O'Halloran DM, Martin CM. Understanding health system reform - A complex adaptive systems perspective. *J Eval Clin Pract* 2012;**18**:202–8. doi:10.1111/j.1365-2753.2011.01792.x

29 Sturmberg JP. Want improved quality? Improve your systems. *J Eval Clin Pract* 2020;**26**:1530–3. doi:10.1111/jep.13461

30 Stulberg JJ, Schäfer WLA, Shallcross ML, *et al.* Evaluating the implementation and effectiveness of a multi-component intervention to reduce post-surgical opioid prescribing: Study protocol of a mixed-methods design. *BMJ Open* 2019;**9**:1–11. doi:10.1136/bmjopen-2019-030404

31 Grove A, Clarke A, Currie G, *et al.* Advancing clinical leadership to improve the implementation of evidence-based practice in surgery: a longitudinal mixed-method study protocol. *Implement Sci* 2020;**15**:1–10. doi:10.1186/s13012-020-01063-2

32 Ha NHL, Chan I, Yap P, *et al.* Mixed-method evaluation of CARITAS: A hospital-To-community model of integrated care for dementia. *BMJ Open* 2020;**10**:1–12. doi:10.1136/bmjopen-2020-039017

33 Schmidt B, Watt K, McDermott R, *et al.* Assessing the link between implementation fidelity and health outcomes for a trial of intensive case management by community health workers: A mixed methods study protocol. *BMC Health Serv Res* 2017;**17**:1–6. doi:10.1186/s12913-017-2320-2

34 Mann R, Beresford B, Parker G, *et al.* Models of reablement evaluation (MoRE): A study protocol of a quasi-experimental mixed methods evaluation of reablement services in England Organization, structure and delivery of healthcare. *BMC Health Serv Res* 2016;**16**:1–9. doi:10.1186/s12913-016-1600-6

35 Manojlovich M, Adler-milstein J, Harrod M, *et al.* The Effect of Health Information Technology on Health Care Provider Communication: A Mixed-Method Protocol. *JMIR Res Protoc*

For peer review only

2015;**4**:e72. doi:10.2196/resprot.4463

36 Allan S, Mcleod H, Bradstreet S, *et al.* Understanding Implementation of a Digital Self-Monitoring Intervention for Relapse Prevention in Psychosis: Protocol for a Mixed Method Process Evaluation. *JMIR Res Protoc* 2019;**8**:e15634. doi:10.2196/15634

37 Sun Y, Luo R, Li Y, *et al.* App-Based Salt Reduction Intervention in School Children and Their Families (AppSalt) in China Protocol for a Mixed Methods Process. *JMIR Res Protoc* 2021;**10**:e19430. doi:10.2196/19430

38 Ward M, McAuliffe E, Wakai A, *et al.* Study protocol for evaluating the implementation and effectiveness of an emergency department longitudinal patient monitoring system using a mixed-methods approach. *BMC Health Serv Res* 2017;**17**:1–10. doi:10.1186/s12913-017-2014-9

39 Farre A, Cummins C. Understanding and evaluating the effects of implementing an electronic paediatric prescribing system on care provision and hospital work in paediatric hospital ward settings: A qualitatively driven mixed-method study protocol. *BMJ Open* 2016;**6**:1–9. doi:10.1136/bmjopen-2015-010444

40 Van Huizen LS, Dijkstra P, Halmos GB, *et al.* Does multidisciplinary videoconferencing between a head-and-neck cancer centre and its partner hospital add value to their patient care and decision-making? A mixed-method evaluation. *BMJ Open* 2019;**9**. doi:10.1136/bmjopen-2018-028609

41 Department of Health. Research governance framework for health and social care. *Health Soc Care Community* 2005;**10**:1–54.

42 Weiskopf NG, Bakken S, Hripcsak G, *et al.* A data quality assessment guideline for electronic health record data reuse. *J Electron Heal Data Methods* 2017;**5**:1–19.https://egems.academyhealth.org/articles/10.5334/egems.218/

43 Weiskopf NG, Bakken S, Hripcsak G, *et al.* Appendix B_A data quality assessment guideline for electronic health record data reuse. *J Electron Heal Data Methods* 2017;**5**:1–19. doi:10.13063

44 Fernandez-Llatas C, Munoz-Gama J, Martin N, *et al.* Process Mining in Healthcare. In: Fernandez-Llatas C, ed. *Interactive Process Mining in Healthcare. Health Informatics*. Champaign, IL: : Springer 2021. doi:10.1007/978-3-030-53993-1_4

45 Mans RS, van der Aalst WMP, Vanwersch RJB. *Process Mining in Healthcare: Evaluating and Exploiting Operational Healthcare Processes*. Springer 2015. doi:10.1007/978-3-319-16071-9

46 Mans RS, Reijers HA, Van Genuchten MJIM, *et al.* Mining Processes in Dentistry. In: *IHI'12: proceedings of the 2nd ACM SIGHIT: International Health Informatics Symposium*. New York: : ACM 2012. 379–88.

47 Baker K, Dunwoodie E, Jones RG, *et al.* Process mining routinely collected electronic health records to define real-life clinical pathways during chemotherapy. *Int J Med Inform* 2017;**103**:32–41. doi:10.1016/j.ijmedinf.2017.03.011

48 Mannhardt F, Blinde D. Analyzing the trajectories of patients with sepsis using process mining. In: *CEUR Workshop Proceedings*. 2017. 72–80.http://ceur-ws.org/Vol-1859/bpmds-08-paper.pdf

49 Williams R, Ashcroft DM, Brown B, *et al.* Process mining in primary care: Avoiding adverse events due to hazardous prescribing. *Stud Health Technol Inform* 2019;**264**:447–51. doi:10.3233/SHTI190261

For peer review only

50 Van der Aalst W, Adriansyah A, Van Dongen B. Replaying history on process models for conformance checking and performance analysis. *Wiley Interdiscip Rev Data Min Knowl Discov* 2012;**2**:182–92. doi:10.1002/widm.1045

51 Kurniati AP, McInerney CD, Zucker K, *et al.* A multi-level approach for identifying process change in cancer pathways. In: *Conference: 17th International Conference on Business Process Management (BPM 2019)*. Vienna, Austria: 2019.

52 McDonald KM, Romano PS, Geppert J, *et al. Measures of Patient Safety Based on Hospital Administrative Data - The Patient Safety Indicators*. Agency for Healthcare Research and Quality 2002.

53 Greenhalgh T, Swinglehurst D. Studying technology use as social practice: the untapped potential of ethnography. *BMC Med* 2001;:45.

54 Holloway I. *Qualitative research in health care*. Maidenhead: : OUP 2005.

55 McDonald S. Studying actions in context: a qualitative shadowing method for organizational research. *Qual Res* 2005;**5**:455–73.

56 Flanagan J. The Critical Incident Technique. *Psychol Bull* 1954.

57 Moore GF, Audrey S, Barker M, *et al.* Process evaluation of complex interventions: Medical Research Council guidance. *BMJ* 2015;**350**.

58 Greenhalgh T, Wherton J, Papoutsi C, *et al.* Beyond adoption: A new framework for theorizing and evaluating nonadoption, abandonment, and challenges to the scale-up, spread, and sustainability of health and care technologies. *J Med Internet Res* 2017;**19**. doi:10.2196/jmir.8775

59 Kulak D, Guiney E. *Use Cases: Requirements in Context*. Addison-Wesley 272 2012.

60 Randell R, Dowding D. Organisational influences on nurses' use of clinical decision support systems. *Int J Med Inform* 2010;**79**:412–21. doi:10.1016/j.ijmedinf.2010.02.003

61 Ritchie J, Spencer L. Qualitative data analysis for applied policy research. In: Bryman A, Burgess E, eds. *Analysing qualitative data*. Oxon: : Routledge 1994.

62 Endsley MR. Toward a Theory of Situation Awareness in Dynamic Systems. *Hum Factors* 1995;**37**:32–64.

63 Faraj S, Xiao Y. Coordination in Fast-Response Organizations. *Manage Sci* 2006;**52**:1155–69.

64 Weick KE, Sutcliffe KM. *Managing the Unexpected: Assuring High Performance in an Age of Uncertainty*. 2nd ed. San Francisco, CA: : Wiley 2001.

65 Jordan S, Johannessen I. Mindfulness and Organizational Defenses: Exploring Organizational and Institutional Challenges to Mindfulness. In: Ngnoumen A, Langer E, eds. *The Wiley Blackwell Handbook of Mindfulness*. John Wiley & Sons 2014. 424–42.

66 Holden RJ, Karsh B-T. The Technology Acceptance Model: Its past and its future in health care. *J Biomed Inform* 2010;**43**:159–72.

67 Roberts KH, Madsen P, Desai V, *et al.* A case of the birth and death of a high reliability healthcare organisation. *Qual Saf Heal Care* 2005;**14**:216–20. doi:10.1136/qshc.2003.009589

68 Benn J, Healey AN, Hollnagel E. Improving performance reliability in surgical systems. *Cogn Technol Work* 2008;**10**:323–33. doi:10.1007/s10111-007-0092-x

For peer review only

69 Hollnagel E. Resilience engineering and the systemic view of safety at work: Why work-as-done is not the same as work-as-imagined. *Bericht zum 58 Kongress der Gesellschaft für Arbeitswiss vom* 2012;**22**:19–24.

70 Benn J, Koutantji M, Wallace L, *et al.* Feedback from incident reporting: Information and action to improve patient safety. *Qual Saf Heal Care* 2009;**18**:11–21.

71 Research BI for H. Connected Yorkshire. 2021.https://www.bradfordresearch.nhs.uk/our-research-teams/connected-bradford/

For peer review only

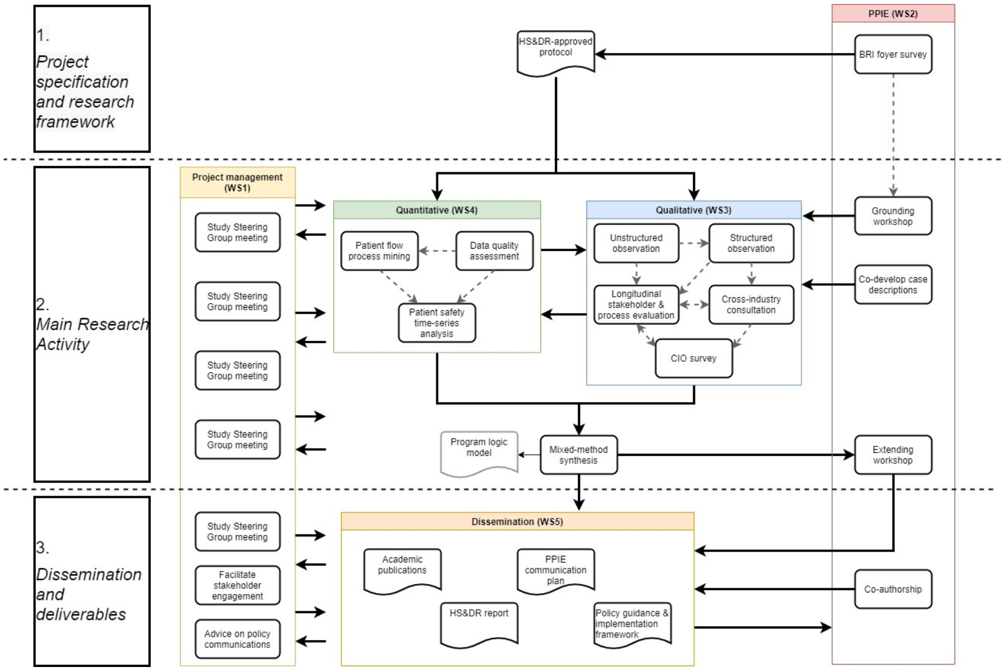

460x304mm (72 x 72 DPI)

Research Aim-Activities Matrix

Below is a matrix that indicates which research activities address which research aims and objectives

				Patient-safety time-series analysis	Patient-flow process mining	Data quality assessment	Unstructured observation	Structured observation	Stakeholder interview	Cross-industry consultation	CIO survey	Mixed-method synthesis	Dissemination
**Aim 1**	Evaluate the impact of the CC on patient safety, hospital operational efficiency and related organisational processes.	**a**	Describe (qualitatively) and evaluate (statistically) any effect on patient safety, including monitoring of deteriorating patients and sub-optimal care pathways, risk of harm due to cancellation/delays and situational awareness in safety-critical areas such as the emergency department.	X			X	X	X				
		**b**	Describe (qualitatively) and evaluate (statistically) any effect on patient flow, including capacity-demand ratio, transfer delays, bed utilisation, timely discharge and cancellations of scheduled care.		X		X	X	X				
		**c**	Qualitatively investigate any effect on organisational processes, such as situational awareness, operational decision-making, risk and coordination/communication across organisational units, from multiple stakeholder perspectives.				X	X	X				
**Aim 2**	Understand the process of implementation and integration of the CC and associated data infrastructure and organisational processes within the primary study site	**a**	Using qualitative methods, describe the process of development and implementation of the CC, including critical implementation factors and any unintended consequences.				X	X	X				
		**b**	Through ethnographic methods, investigate the process by which the CC system and outputs are embedded at all levels of the organisation, from frontline operations to strategic quality and safety governance.					X					
		**c**	Develop and validate a logic model for this health informatics intervention that maps system preconditions, processes, technology and outcomes, at the primary study site.									X	
		**d**	Describe (statistically and qualitatively) the effect of the CC implementation on the local data environment, including data infrastructure, quality and integration (i.e. system interoperability).			X	X	X	X				
**Aim 3**	Elicit cross-sector and cross-industry perspectives on hospital command and control technologies to contextualise the findings from the	**a**	Review and understand command and control processes in non-healthcare safety-critical operations and the key principles and contextual factors that may influence transferability of these models into a hospital setting.							X			

For peer review only - http://bmjopen.bmj.com/site/about/guidelines.xhtml

	primary study site for broader application.	**b**	Survey the perceptions of senior health informatics professionals on current command and control processes, viability of novel “mission-control” systems, data readiness and potential implementation barriers.								X		
**Aim 4**	Synthesise the research findings into practical outputs that will engage service stakeholders and inform future investment and practice	**a**	Share learning concerning cross-industry and empirical findings on the costs-benefit of investment within NHS management and Chief Information Officer networks.										X
		**b**	Construct an empirically-informed implementation framework that describes contextual factors and implementation pathway for development of centralised, data-driven mission-control systems in acute care, including data infrastructure maturity.									X	

For peer review only

Study Steering Group membership

Below are the details of the members of the Study Steering Committee for the NIHR HS&DR project entitled "Evaluating the safety and patient impacts of an AI Command Centre in the NHS" (NIHR129483).

Name	Affiliation	Reason for nomination
Iain Buchan (Chair)	University of Liverpool; University of Manchester (Honorary)	Executive Dean, Institute of Population Health Sciences; Director of Digital Strategy and Partnerships, Liverpool Health Partners; Chair of Public Health and Clinical Informatics
Paul Charnley	Wirral NHS Teaching Hospitals Trust	Director of IT and Information at Wirral NHS Teaching Hospitals Trust
Sarah Culkin	NHS England; NHSX	Artificial Intelligence lead for NHS England
Cindy Fedell	Regional Chief Information Officer at Northwestern Ontario Hospitals	Former Chief Information Officer at Bradford Teaching Hospitals NHS Foundation Trust
Hamish Fraser	Brown University, USA	A health informatics academic and clinical lead for OpenMRS, the most-widely used open-source medical record system
Charles Koo	Envive Technology, Ltd.; Visiting Scholar (Stanford University); Visiting Expert (National Taiwan University)	Implemented large scale Artificial-Intelligence, hospital systems at six hospitals in Shanghai, China.
Farah Magrabi	Macquarie University	Associate Professor Australian Institute of Health Innovation
Sue Mason	Sheffield University; Sheffield NHS Teaching Hospitals Trust	Professor of Emergency Medicine at Sheffield University and clinician at Sheffield NHS Teaching Hospitals Trust
Mark Sujan	University of Warwick; Human Factors Everywhere	Human Factors expert
Hilary Thompson	NIHR Yorkshire and Humber Patient Safety Translational Research Centre	Patient representative and NIHR YH PSTRC Lay Leader
Sean White	NHS Digital	Senior Safety Engineer
Patrick Waterson	Loughborough University	Reader in Human Factors and Complex Systems

For peer review only

Research Data Leeds http://researchdata.leeds.ac.uk/ email: researchdataenquiries@leeds.ac.uk
Oct 2019

University of Leeds Data Management Plan (DMP) Template

Researcher Name	Owen Johnson
Project Title	Evaluating the safety and patient impact of an Artificial Intelligence Command Centre in the UK National Health Service
Faculty	Engineering and Physical Sciences
KRISTAL Reference Number (if applicable)	118684
Supervisor(s) name (if applicable)	n/a
Funder	National Institute for Health Research
Scheme	
Research Start Date	1st March 2021
Research End Date	30th Sept 2022
Ethical review number	Not available, yet
DMP review due	3rd May 2021

Date	Version	Author	Change notes
11th Nov 2020	V1.0	Ciarán McInerney	n/a
16th Dec 2020	V2.0	Ciarán McInerney and Carolyn McCrorie	Added data management plan for qualitative work.
10th Feb 2021	V3.0	Ciarán McInerney and Carolyn McCrorie	Added details of the standards with which Yorkshire and Humber Care Record's Google Cloud Platform are compliant.

Please provide a brief overview of your project including proposed research methods

AI Command Centre in Bradford NHS Bradford Teaching Hospitals NHS Foundation Trust is implementing an artificial intelligence (AI) command centre in Bradford Royal Infirmary, which is regarded as the first of its kind in Europe. The command centre follows an approach successfully used in the USA (Johns Hopkins, Baltimore) and Canada (Humber River, Toronto) to provide real-time, rapid response to clinical, management and patient-flow challenges. Similar to an air traffic control command centre, hospital staff work together in a purpose-built operations room and monitor a 'wall of analytics' of high-definition screens that display real-time data from the hospital's clinical systems. The team review, monitor and react to the 'big picture' of how efficiently patients are flowing through the hospital, where bottlenecks might occur, where pressure is building and where safety breaches are predicted. The command centre software makes use of AI technologies that are refined through operation to provide increasingly more intelligent alerts and warnings. There is a very limited evidence base for this form of digital technology in hospitals but an increasing belief that AI can and should play a key role in transforming and modernising the NHS. Our research team in the NIHR Yorkshire & Humber Patient Safety Translational Research Centre (YH-PSTRC) are based in Bradford and in a unique position to collect and study the evidence. Research Framework We propose a mixed-method study with three phases over 18 months, commencing in 2020. In the first phase, we will carry out initial scoping work with relevant stakeholders. The second and third phases will involve longitudinal case studies describing the impact of an AI command centre on safety. We will also

For peer review only - http://bmjopen.bmj.com/site/about/guidelines.xhtml

Research Data Leeds http://researchdata.leeds.ac.uk/ email: researchdataenquiries@leeds.ac.uk
Oct 2019

conduct a mixed-method comparison with hospitals such as Calderdale and Huddersfield NHS foundation Trust, which has shared systems and learning through close collaboration with Bradford. Outputs Our aim in this study is to provide a robust academic evaluation of the AI Command Centre so that other hospitals and healthcare providers can consider how best to exploit this emerging technology. The findings from our study will be reported to the NHS trusts, including local dissemination of findings. They will also be reported in a white paper for distribution to NHS Chief Information Officers, in academic publications, to NIHR as part of the annual report for funding body for the YH-PSTRC and as funders for this specific project and dissemination of findings via the NIHR PSTRCs' networks.

1. What data will be produced? What data will be used from other sources?

With respect to the quantitative component of the project:

1. Study on patient safety.
Collect:

- **What?** - Routinely-collected electronic health record data that informs the measures of patient safety identified during early qualitative work. This data is created and stored in Bradford Teaching Hospital NHS Trust's electronic health record system, Cerner Millennium. This data will be de-identified and aggregated within the Yorkshire Health and Care Record, from which we will request extracts.
- **Why?** - This data is created by the hospital system being studied and so reflects the safety performance of the hospital system.

Create:

- **What?** - Summary statistics of the distribution and dynamics of patient-safety measures at both sites. Visualisations of the values. Models of variable dynamics. We will also create R scripts, which are text documents containing instructions to run commands in the R statistical programme.
- **Why?** - The measures of patient-safety are the variables of interest in this study. The R scripts are needed to process the data. The visualisations will facilitate investigations and some will be used to communicate the research.

2. Study on patient flow.
Collect:

- **What?** - Routinely-collected electronic health record data created and stored in Bradford Teaching Hospital NHS Trust's electronic health record system, Cerner Millennium. This data will be de-identified within the Yorkshire Health and Care Record, from which we will request extracts.
- **Why?** - Pseudonymised patient identifier, a description of a clinical event, and the timestamp for the event are the minimal requirements to build a process model of a patient flow through a hospital.

Create:

Adapted from University of Cambridge DataTrain project & University of Edinburgh MANTRA project

Research Data Leeds http://researchdata.leeds.ac.uk/ email: researchdataenquiries@leeds.ac.uk
Oct 2019

- **What?** - We will create R scripts, which are text documents containing instructions to run commands in the R statistical programme. These R scripts will produce R files containing process models and summary statistics describing the process models. Visualisations of the models and values.
- **Why?** - The models describe patient flow, which is a marker of patient safety. These models need to be evaluated so we compute summary statistics of their performance. The R scripts are needed to process the data. The visualisations will facilitate investigations and some will be used to communicate the research.

3. Study on data quality.
Collect:

- **What?** - Routinely-collected electronic health record data that are presented or used to inform variables that are presented on the AI Command Centre tiles. This data is created and stored in Bradford Teaching Hospital NHS Trust's electronic health record system, Cerner Millennium. Identifying the variables of interest will require an audit of the tiles of interest, which will be conducted in early qualitative work.
- **Why?** - This data is created by the hospital system being studied. We wish to assess how the quality of this data might have changed as the Command Centre was gradually implemented.

Create:

- **What?** - We will be using the Weiskopf et al. (2017) tool for assessing the quality of the data, which will create the data indicated in the table below, as per their 3x3 data-quality matrix. Each cell of the matrix will inform a visualisation of the data. We will also create R scripts, which are text documents containing instructions to run commands in the R statistical programme.

	Complete	*Correct*	*Current*
Patients	Counts and percentages of Variables, Times with recorded data, and Overall points of data present.	Details of expected value limits for the variables across patients. A citation for the source of the expectation. Counts and percentages of patients whose data are within the value limits.	Details of the expected period within which the data were recorded. A citation for the source of the expectation. For each non-static variable, Counts and percentages of patients whose data do not fall within the desired date range. Summary statistics of the discrepancies between actual recording dates and desired date ranges (mean, median, mode, 1st quartile, 3rd quartile, interquartile range, minimum,

For peer review only - http://bmjopen.bmj.com/site/about/guidelines.xhtml

			maximum, standard deviation, variance)
Variables	Counts and percentages of Patients with the variable, Times with the variable present, and Overall points of data present.	Details of criteria that indicate concordance for each variable. A citation for the source of the expectation. Counts and percentages of patients who meet each criterion. Counts and percentages of patients whose data violate any of the criteria.	Details of the expected sequence or intervals between events. A citation for the source of the expectation. Counts and percentages of patients that meet expectations, for each variable. Counts and percentages of patients that meet all expectations.
Times	Counts and percentages of Patients with data recorded for that time, Variables with data recorded for that time, and Overall points of data present.	Details of criteria that indicate valid changes over time, for each variable. A citation for the source of the expectation. Counts and percentages of patients who meet each criterion. Counts and percentages of patients whose data violate any of the criteria.	The I-score for each variable for which regularity is to be assessed. Summary statistics for the I-score across all variables (mean, median, mode, 1st quartile, 3rd quartile, interquartile range, minimum, maximum, standard deviation, variance). The IxN-score to measure "effective data points".

- **Why?** - The data created using the Weiskopf et al. (2017) tool for assessing the quality are the variables of interest in this study. The R scripts are needed to process the data. The visualisations will facilitate investigations and some will be used to communicate the research.

With respect to the qualitative component of the project:

1. Case description and unstructured observation

Collect:

- **What?** -Ethnographic observations within the Command Centre (CC) Unit. Up to 36 hours over 4-hour periods.
- **Why?** -In order to immerse and sensitise the research team to the context of hospital operational command and control. The data will help us to understand events and actions as they unfold from the actor's perspective (and the meanings that CC users attach to them).

Create:

- **What?** -Researcher field notes

Adapted from University of Cambridge DataTrain project & University of Edinburgh MANTRA project

For peer review only - http://bmjopen.bmj.com/site/about/guidelines.xhtml

- **Why?** -To draw upon the concepts of Grounded Theory in which we will adopt an inductive qualitative analysis approach to understand way in which the CC system integrates within the broader hospital information and operational planning systems in a formal model grounded in our data.

2. System-wide structured observation

Collect:

- **What?** -Ethnographic observations within and beyond the Command Centre. Up to 10 hours observation each of specific tracer issues/professional roles that represent interaction with CC processes and outputs.
- **Why?** -In order to explore the impact of the CC beyond the operations room and at all levels of the organisation, including micro-level (frontline clinical workflow in specific specialties), meso-level operational planning (e.g. bed management) and macro-level strategic planning (e.g. use of data in quality and safety governance).

Create:

- **What?** -Researcher field notes
- **Why?** -To draw upon concepts of Realist Evaluation in our analysis to understand usability of the system in context.

3. Longitudinal stakeholder and process evaluation

Collect:

- **What?** -Qualitative research interviews with up to 24 staff relative to the initiative
- **Why?** -To evaluate the efficacy of the system from multiple user perspectives

Create:

- **What?** -Interview transcripts
- **Why?** -To draw upon a process evaluation framework approach to analysis in order to understand intervention mechanisms, implementation processes, interaction with context and overall outcomes.

4. Cross-industry study

Collect:

- **What?** -Qualitative research interviews with up to 10 consultants in safety critical industries
- **Why?** -To elicit and apply knowledge from high-risk industry to the development of strategies for implementing command and control centres to improve quality and safety

Create:

- **What?** -Interview transcripts

Adapted from University of Cambridge DataTrain project & University of Edinburgh MANTRA project

For peer review only - http://bmjopen.bmj.com/site/about/guidelines.xhtml

- **Why?** -To draw upon a process evaluation framework approach to analysis in order to understand intervention mechanisms, implementation processes, interaction with context and overall outcomes.

5. Survey study

Collect:

- **What?** -Survey of a sample of Chief Information Officers in acute care across England and Wales
- **Why?** -To capture views on current practices in data-supported operational planning

Create:

- **What?** -Survey responses captured through UoL Online Surveys
- **Why?** -The data is required to understand variations in electronic data-facilitated command and control beyond the 2 research sites

2. Where will data be stored? How will data be structured? Include file formats and approximate volume.

Where will data be stored?

The electronic health records used in the quantitative work will not be copied for storage or back-up, by the research team. Instead, it will be hosted by the Yorkshire and Humber Care Record and accessed via their virtual research environment. The Yorkshire and Humber Care Record system is built on Google Cloud technology with Identity Access Management following the principle of least privilege, i.e. minimum permissions of access and functionality. Google Cloud technology is compliant with GDPR, ISO/IEC 27001, ISO/IEC 27017, ISO/IEC 27018, ISO/IEC 27701, NHS Digital Commercial Third-Party Information Governance Requirements, UK's Cloud Security Principles. Further details are available at https://cloud.google.com/security/compliance.

Toward the end of the project, summative research output for publications and all R scripts used for data processing will be exported from the Yorkshire and Humber Care Record portal and stored on the University of Leeds SAN (Storage Area Network), which comprises enterprise level disk storage and file servers located in physically secure data centres with appropriate fire suppression equipment. Snapshots are taken every day at 10pm (and accessible for 1 month). A second level of snapshots is taken every month and are kept for 11 months. Snapshots are user recoverable from the desktop.

A full back-up to tape is taken once every month and an incremental copy to backup tape is taken every night (and kept for 28 days). Every quarter, the most recent set of full dump tapes are moved to a long-term storage facility where they are kept for 12 months. Tapes are initially stored in on-campus fireproof safes and then moved to off-campus secure locations. The SAN is located behind the University's Institutional firewall to protect against external attacks.

During the life of the qualitative work, the data will be stored on the University of Leeds SAN. The audio-recording equipment will be encrypted. Survey data will be stored in UoL Online Surveys

After the project has completed, data will be offered to the University of Leeds Research Data Repository (Research Data Leeds) or another appropriate data repository service in order to ensure the data can be shared, reused and cited beyond the end of the project. Research Data Leeds holds deposited data for a minimum of 10 years and datasets are associated with digital object identifiers (DOIs).

How will data be structured?

For peer review only - http://bmjopen.bmj.com/site/about/guidelines.xhtml

With respect to the 'collected' quantitative data (in so far as 'data collection' refers to the method by which data is obtained):

- **Format** - The data is expected to be accessible in the Yorkshire Health and Care Record portal as a CSV file or in one of the SQL database file types, which we would subsequently transform into a CSV file type.
- **Volume** - The size of the dataset will be informed mostly by the count of patients, rather than by the count of variables. Both hospital sites under study typically see in the range of 70,000 - 80,000 unique patients every month, each with at least two events (admission and discharge). This translates to a volume of data in the order of millions of observations across multiple variables. File sizes are likely to be in the order of MBs.

With respect to the created quantitative data:

- **Format** - The data will be stored as R files and CSV files, outputted using RStudio. The R format will facilitate analysis while the CSV formats are preferred to transferability to other software. All visualisations will be stored in PNG and JPEG formats. The JPEG formats are have smaller file sizes and will be used only when the higher quality PNG format is not supported.
- **Volume** - Likely no more than low double figures of megabytes.

With respect to the collected qualitative data:

- **Format** – The interview transcripts and field notes will be stored as files within NVivo 12 version. Survey data will be stored in SPSS software.
- **Volume** – The size of the data set will be informed by the number of interviews undertaken, size of the field notes and number of responses to the survey.

With respect to the created qualitative data:

- **Format** - The interview transcripts and field notes will be stored as files within NVivo 12 version. Survey data will be stored in SPSS software.
- **Volume** – The size of the data set will be informed by the number of interviews undertaken, size of the field notes and number of responses to the survey.

3. Access to data during the project. Give details of collaborators and any controls.

During the life of the quantitative work, access to the 'collected' data will be controlled by the Yorkshire and Humber Care Record, with whom our research team will have a contract stipulating the terms of use of the Yorkshire and Humber Care Record portal. The created data that will occasionally be exported and stored on University of Leeds' Storage Area Network. These data will only be accessible by university staff with user privileges and password access.
During the life of the qualitative work, the data will be stored on UOL SAN. Only members of the research team will have access via user privileges and password access.
After the project has completed, the data that will be stored with the data repository service will only be accessible on request and following approval criteria that will be co-developed by the research team and the data repository service.

4. Ethics and legal compliance: are there any 'special' requirements for your data? Any contractual or consent issues? Key policies (internal and external)

Adapted from University of Cambridge DataTrain project & University of Edinburgh MANTRA project

For peer review only - http://bmjopen.bmj.com/site/about/guidelines.xhtml

Research Data Leeds http://researchdata.leeds.ac.uk/ email: researchdataenquiries@leeds.ac.uk
Oct 2019

Consent – all interviewees will give informed consent for the interview to be audio-recorded and transcribed.
5. How will data be documented and described? Methodologies and protocols.<br>All folders will contain a README in a TXT file format that explains what files are in the folder. Each file will be named, its provenance (including source and steps taken to process it), and details of any restrictions on sharing. No formal standard will be adhered to.
6. Training and support<br><br>All researchers have completed UoL training in Information Security Essential.
7. What are the plans for data sharing beyond project partners? Include justification if some of your data needs to be restricted. Include data and code. Include repository.<br><br>After the project has completed, the data that will be stored with a data repository service will only be accessible on request and following approval criteria that will be co-developed by the research team and the data repository service. Data will be made available via the University of Leeds data repository and, where possible, as supplementary material accompanying academic publications.<br>The electronic health records used in the quantitative work will not be made available outside of the Yorkshire and Humber Care Record in which it was accessed.
8. What Intellectual Property will be generated? How will IP be protected and exploited?<br>We are not expecting to generate Intellectual Property beyond the academic outputs produce vai the research process.
9. Who is responsible for managing the data? What resources will you need?<br><br>The Principal Investigator will have ultimate responsibility for data management during the project but the quantitative and qualitative study leads will have day-to-day responsibilities. After the project, the data will be managed by the University of Leeds data repository staff.
10. Ongoing data curation / data housekeeping - you may find it useful to include a retention table<br><br>All summative data used to communicate research findings in published output will be stored in the University of Leeds data repository. This repository holds deposited data for a minimum of 10 years and datasets are associated with digital object identifiers (DOIs).

End of Project

At the end of a project and/or before you leave the institution, you should ensure that data and research materials are deposited with the School or a trusted data repository and documented in such a way that they can be found and understood.

Dataset name	Location	Person responsible

CC BY NC SA

For peer review only - http://bmjopen.bmj.com/site/about/guidelines.xhtml

Research Data Leeds http://researchdata.leeds.ac.uk/ email: researchdataenquiries@leeds.ac.uk

Oct 2019

University of Leeds Data Management Plan (DMP) Template: Prompt Sheet

1. What data will be produced or used? (Including original software)

- ☐ What physical data will you study? (e.g. artefacts, samples, paper archives, etc.)
- ☐ What digital data will you generate? (e.g. field-notes, images, spreadsheets, audio interviews, survey data, annotated bibliography, etc.)
- ☐ What original software will you generate?
- ☐ What third party data will you reuse?

2. Where will data be stored? How will data be structured?

- ☐ Estimate how much data you will produce over time – do you have enough storage?
- ☐ Do you know what University storage is available and how to access it?
- ☐ What file formats and software will you use?
- ☐ Do you have a logical file naming convention and directory structure?
- ☐ How will you use versioning so you can identify the current version of documents / data?
- ☐ How will data generated in the field be saved to safe University storage?

3. Access to data during the project. Give details of collaborators and any controls.

- ☐ Have you discussed data sharing with your research collaborators/ supervisor?
- ☐ Who needs to access data during the research? How will they access data?
- ☐ Do you need a data sharing agreement? (see also section 4.)

4. Ethics and legal compliance: are there any 'special' requirements for your data?

- ☐ Have you read the University's Information Protection Policy? Data must be assessed for sensitivity and storage in line with this policy https://it.leeds.ac.uk/it?id=kb_article&sysparm_article=KB0011140
- ☐ Are you familiar with the University's advice on data protection and GDPR? https://dataprotection.leeds.ac.uk/
- ☐ Does your research funder have specific data management and sharing requirements?
- ☐ Are there other policies and protocols you need to be aware of and observe? For example, NHS codes of practice?
- ☐ Will you anonymise your data?
- ☐ Should some data be destroyed? When and how?
- ☐ How and where will you record any participant consents and/or contractual requirements which impact data management and sharing? The DMP can be a good place to record this information.

5. How will data be documented and described? Methodologies and protocols.

- ☐ Will others understand your data? Write documentation. Make sure table and spreadsheet values are clearly labelled.
- ☐ What information about data collection methodology will be recorded?
- ☐ Is it important for the research to be reproducible? Why/why not? What additional documentation will be required?
- ☐ Will you write software? Where will this be documented and stored for future use?

Adapted from University of Cambridge DataTrain project & University of Edinburgh MANTRA project

For peer review only - http://bmjopen.bmj.com/site/about/guidelines.xhtml

Research Data Leeds http://researchdata.leeds.ac.uk/ email: researchdataenquiries@leeds.ac.uk
Oct 2019

6. Training and support

- ☐ What training do you need for data gathering, organisation, analysis or presentation?
- ☐ Are there relevant courses available at the University? Online? Who can provide support?

7. What are the plans for data sharing beyond project partners?

- ☐ Have you considered reasons for and against sharing data? Will data be openly available to everyone or will there be access restrictions?
- ☐ If your research involves people, have you obtained appropriate consent for data sharing?
- ☐ Can your data be released immediately, or should you embargo (delay access to) the data?
- ☐ How long will / should data be available for?
- ☐ Will you use a data repository? Which one? Are there subject specific data repositories in your field?

8. What IPR will be generated? How will IPR be protected and exploited?

- ☐ Will you be applying for a patent? Will your research have commercial applications? Do you need to contact the Commercialisation team in the Research and Innovation Service?
- ☐ Have you read the University Intellectual Property Policy? http://ris.leeds.ac.uk/downloads/download/600/university_of_leeds_ipr_policy

9. Who is responsible for managing the data? What resources will you need?

- ☐ Who is responsible for data at different stages in its lifecycle?
- ☐ On projects with complex data management requirements, different types of role should be specified.
- ☐ How will best practice and guidance be shared across the project partners?
- ☐ Are sufficient resources (skills, people, storage, technology) available to deliver your plan?

10. Ongoing data curation / data housekeeping - you may find it useful to include a retention table

- ☐ What data will you keep? Who decides?
- ☐ Where will data be kept and for how long.
- ☐ Who needs to know what data exists on the network, where it is, how it should be managed and how long it should be retained?

For peer review only

Don't forget to review and update your data management plan regularly

---

But I don't have any data! *Anything can become research data if it is used for research purposes – data is not just numbers on a spreadsheet. Think creatively about the materials you are using and producing: what could be shared with other researchers who are interested in your work; what could be reused to produce new insights? Any evidence or material which underpins or sheds light on your findings, your academic publications, your thesis or your project can be considered research data.*

Adapted from University of Cambridge DataTrain project & University of Edinburgh MANTRA project

CC BY NC SA

BMJ Open

Evaluating the safety and patient impacts of an Artificial Intelligence Command Centre in acute hospital care: A mixed-methods protocol

Journal:	*BMJ Open*
Manuscript ID	bmjopen-2021-054090.R1
Article Type:	Protocol
Date Submitted by the Author:	15-Nov-2021
Complete List of Authors:	McInerney, Ciarán ; University of Leeds Faculty of Engineering and Physical Sciences, School of Computing; Wolfson Centre for Applied Health Research, Bradford Royal Infirmary<br>McCrorie, Carolyn; University of Leeds Faculty of Social Sciences, School of Psychology; Wolfson Centre for Applied Health Research, Bradford Royal Infirmary<br>Benn, Jonathan; University of Leeds Faculty of Social Sciences, School of Psychology; Wolfson Centre for Applied Health Research, Bradford Royal Infirmary<br>Habli, Ibrahim; University of York, Department of Computer Science<br>Lawton, Tom; Bradford Teaching Hospitals NHS Foundation Trust, Bradford Royal Infirmary<br>Mebrahtu, Teumzghi; University of Leeds Faculty of Engineering and Physical Sciences, School of Computing<br>Randell, Rebecca; University of Bradford, Faculty of Health Studies; Wolfson Centre for Applied Health Research, Bradford Royal Infirmary<br>Sheikh, Naeem; Wolfson Centre for Applied Health Research, Bradford Royal Infirmary<br>Johnson, Owen; University of Leeds Faculty of Engineering and Physical Sciences, School of Computing; Wolfson Centre for Applied Health Research, Bradford Royal Infirmary
**Primary Subject Heading**:	Health informatics
Secondary Subject Heading:	Health informatics
Keywords:	Health informatics < BIOTECHNOLOGY & BIOINFORMATICS, Information management < BIOTECHNOLOGY & BIOINFORMATICS, Information technology < BIOTECHNOLOGY & BIOINFORMATICS, Telemedicine < BIOTECHNOLOGY & BIOINFORMATICS

SCHOLARONE™
Manuscripts

Evaluating the safety and patient impacts of an Artificial Intelligence Command Centre in acute hospital care: A mixed-methods protocol

McInerney CD[1 2]*, McCrorie C[2 3], Benn J [2 3], Habli I [4], Lawton T [5], Mebrahtu TF [1], Randell R [2 6], Sheikh N[2], Johnson O [1 2]

[1] School of Computing, University of Leeds, Leeds, UK

[2] Wolfson Centre for Applied Health Research, Bradford Royal Infirmary, Bradford, UK

[3] School of Psychology, University of Leeds, Leeds, UK

[4] Department of Computer Science, University of York, York, UK

[5] Bradford Teaching Hospitals NHS Foundation Trust, Bradford Royal Infirmary, Bradford, UK

[6] Faculty of Health Studies, University of Bradford, Bradford, UK

* **Corresponding author**: Ciarán D. McInerney (c.mcinerney@leeds.ac.uk); School of Computing, Faculty of Engineering and Physical Sciences, University of Leeds, Leeds, LS2 9JT

For peer review only

Abstract

Introduction

This paper presents a mixed methods study protocol that will be used to evaluate a recent implementation of a real-time, centralised hospital command centre in the UK. The Command Centre represents a complex intervention within a complex adaptive system. It could support better operational decision-making and facilitate identification and mitigation of threats to patient safety. There is, however, limited research on the impact of such complex health information technology on patient safety, reliability and operational efficiency of healthcare delivery and this study aims to help address that gap.

Methods and analysis

We will conduct a longitudinal mixed-method evaluation that will be informed by public-and-patient involvement and engagement. Interviews and ethnographic observations will inform iterations with quantitative analysis that will sensitise further qualitative work. Quantitative work will take an iterative approach to identify relevant outcome measures from both the literature and pragmatically from datasets of routinely-collected electronic health records.

Ethics and dissemination

This protocol has been approved by the University of Leeds Engineering and Physical Sciences Research Ethics Committee (#MEEC 20-016) and the NHS Health Research Authority (IRAS No.: 285933). Our results will be communicated through peer-reviewed publications in international journals and conferences. We will provide ongoing feedback as part of our engagement work with local trust stakeholders.

Registration

This study is registered with Research Registry (#researchregistry6611).

WORD COUNT: 4,138

For peer review only

Strengths and limitations of this study

- The study design was informed by patient and public representatives
- The study will use rich electronic health records data and real-time information to assess patient safety and flow
- This is a longitudinal mixed-method evaluation study designed to focus on both outcomes and organisational behaviour, informed by a multidisciplinary co-investigator team.
- The study may be limited in its capability to distinguish between contributions motivated by the response to COVID-19 and the impact of command centre

For peer review only

Introduction

Fragmented healthcare delivery adversely affects patient safety and care.[1,2] Health information technology might help information flow across fragmented organisations[3,4] but investment and adoption has been limited, and is not a completely sufficient solution.[5] The result is a complex socio-technical organisation that challenges healthcare delivery.[6] In the face of dynamic risks and organisational complexity, high-reliability organisations, like nuclear power plants and air traffic control systems, nevertheless demonstrate how safety can be maintained.[7,8]

Such high-reliability organisations use centralised "mission control" structures to integrate intelligence in real time and facilitate safe and efficient operational decision making. Within acute care settings, a hospital "command centre" or "mission control" is a relatively novel concept but one that is now achievable given recent advances in electronic health records, data integration and investment from commercial developers. There is however limited research on the impact of such complex health information technology on patient safety, reliability and operational efficiency of healthcare delivery. It is also unknown whether such centralised structures are appropriate for coordinating a hospital given that a) healthcare provision is not bounded within the hospital, unlike energy provision within a nuclear power plant, b) the system under 'control' is comprised of people, unlike non-living materials in off-shore oil, and c) healthcare operates on a demand-pull dynamic, rather than the supply-push dynamic.

The sociotechnical requirements for effective command centres have been studied in transport and military situations,[9] and there have been attempts to use artificial intelligence (AI) principles within command centres since Project Cybersyn in Chile as early as 1971.[10] There is a limited evidence base for this form of digital technology in healthcare[11] – although some successes have been reported in the USA[12] – but there is an increasing belief, supported by the Government's Life Sciences Strategy, that AI should play a key role in transforming and modernising the NHS in the UK.[13,14] Thus, there

For peer review only

is a need to understand how hospitals manage their operations through advances in health information technology, and how this affects the quality and safety of patient care.

There is a mismatch between the factors required for successful implementation, end users and provider perspectives.[15] The disruptions to workflow are significant challenges for users, particularly in systems that have limited modularity and configurability.[15] There have been very few published attempts to evaluate effectiveness, except for use as a clinical decision support tool[16] and most of the evidence originates from North America.[17] Given the major differences in the social, political and economic foundations of their healthcare system, it is important to explore whether these issues are relevant to the UK context.[18]

Even less is known about the potential of electronic health records developments to improve patient outcomes. Patient journeys are poorly understood, with aggregate statistics obfuscating detail. What has not been achieved is real-time command and control using the data generated by routine systems, despite a rise in the number of state-of-the-art dashboards, flow and simulation models.

Bradford Teaching Hospitals NHS Foundation Trust (BTHFT) provides hospital services for around half a million people in the district of Bradford, West Yorkshire, United Kingdom. In 2019, the Trust implemented a hospital Command Centre, working with commercial suppliers from the USA. Located at the Bradford Royal Infirmary (BRI) hospital, this command centre is made up of GE Kryptonite software and display screens (also known as 'tiles'). The tiles serve various functions according to the needs of the organisation and its patients - some display or summarise information pulled directly from hospital computer systems, while others provide augmented intelligence by interpreting data, such as in the "deteriorating patient" tile which highlights patients at high risk of poor outcomes. Predictive models and automated algorithms generate metrics for display on the tiles in order to provide new insights to support human decision-making, based upon pre-existing data, including information that hospital staff enter into electronic health records.

For peer review only

The Bradford AI Command Centre aims to provide faster and safer care by reducing unnecessary waiting by anticipating and avoiding bottlenecks in care delivery before they cause problems. Such AI command centres have the potential to improve future patient flow and safety, and research to understand the health service delivery, safety and operational factors should be considered an area of major importance for hospitals.

We hypothesise that the implementation and integration of a real-time, centralised hospital command centre will improve patient flow, enhance situational awareness, support operational decision-making and facilitate identification and timely mitigation of threats to patient safety. Supported by funding from the National Institute for Health Research (NIHR), we have proposed a mixed-methods project that combines ethnographic observations, qualitative process evaluation and quasi-experimental methods to study the evolving sociotechnical nature of the systems and processes within hospitals. Our protocol is theoretically informed by contemporary safety science theory concerning system resilience,[19,20] human factors models of situational awareness[21,22] and command-and-control in high reliability organisations.[23–25]

For peer review only

Aim and objectives

Our aim is to evaluate the safety and patient impacts of the Bradford AI Command Centre. Our objectives are:

1. To evaluate the impact of the Command Centre on patient safety, hospital operational efficiency and related organisational processes (‘Impact Evaluation’);
2. To understand the process of implementation and integration of the Command Centre with data infrastructure and organisational processes (‘Process Evaluation’);
3. To contextualise the findings through cross-sector and cross-industry perspectives on hospital command and control technologies (‘Cross-industry perspectives’); *and*

4. To synthesise findings into practical outputs to engage service stakeholders and inform future investment and practice (‘Synthesis and dissemination’).

Further breakdown of the objectives is shown in Appendix 1, indicating the contribution of the research activities described in the next section.

Methods and analysis

Design and data collection

System implementations such the Bradford AI Command Centre are complex interventions into complex adaptive systems that could provide improvements but might also result in emergent unforeseen consequences.[26,27] We will conduct a longitudinal mixed-method evaluation informed by the multidisciplinary co-investigator team and public-and-patient involvement and engagement. A mixed-method approach is well suited to study complexity interventions (e.g. [28]) and the complex adaptive systems to which they are applied. Mixed-method approaches have been used to study information flow and organisational networks,[29] integration of organisational interventions,[30,31] effectiveness of service models,[32] and how health information technology affects communication,[33] patient monitoring,[34–36] care provision,[37] and clinical decision making.[38]

Our study comprises five sub-studies across five work streams (**figure 1**). The five sub-studies are conducted by the qualitative and quantitative work streams (WS3 and WS4 in figure 1). These work streams mutually inform each other as part of an iterative synthesis of findings, rather than solely a summative synthesis. Qualitative and quantitative work streams will work in parallel, with qualitative interviews and ethnographic observations informing iterations for quantitative analysis that will sensitise further qualitative work. Quantitative work will take an iterative approach to identify relevant outcome measures from the literature and pragmatically from the dataset. These will be subsequently verified by the qualitative work.

The main research activity will be guided by the project’s Study Steering Group (see WS1 in figure 1) and by patient-and-public involvement and engagement (PPIE; see WS2 in figure 1). Our Study

For peer review only

Steering Group is an independent body ensuring the project is conducted to the rigorous standards set out in the Department of Health's Research Governance Framework for Health and Social Care and the Guidelines for Good Clinical Practice.[39] The Study Steering Group will also facilitate stakeholder engagement in research and dissemination, and will advise on policy communications (see Ethics and dissemination, below). Membership of our Study Steering Group includes clinical, technical, commercial and academic healthcare representatives from the UK, Canada, USA, China, Australia (Appendix 2).

Sub-study 1: Data quality

This sub-study will contribute to objectives 1 ('Impact Evaluation') and 2 ('Process Evaluation') based on the hypothesis that the introduction of the Command Centre will affect the awareness of, recording of and processing of electronic healthcare data, which are inextricably related to data infrastructure, operational efficiency, and organisational processes. We will apply Weiskopf *et al.*'s 3x3 matrix to assess the quality of healthcare data.[40] This framework maps Patient, Variables and Time data items in terms of Completeness, Correctness and Currency (in other words, presence, accuracy, and timeliness). Further detail on how to implement the 3x3 matrix is available in Weiskopf *et al*.[41] Our initial identification of variables will be informed by our qualitative sub-study (sub-study 4); case-description work will define the Command Centre tiles of interest. The data presented or used to inform variables that are presented on these tiles will then be requested as data abstracts. We will require clinical input to determine the expected attributes of the variables of interest. For example, if a patient has a weekly timestamp in their record but blood pressure is only expected to be taken fortnightly, then we will not consider empty entries every other week as incomplete.

Sub-study 2: Patient flow

This sub-study will contribute to objectives 1 ('Impact Evaluation') and 2 ('Process Evaluation') based on the hypothesis that operational efficiency, organisational processes and patient safety are affected by the flow of patients through the hospital. To study patient flow, we will use process-mining

For peer review only

methods[42] to describe patients' journeys through their hospital care.[43] We will construct process models to represent patients' log of clinical events (see examples in dentistry,[44] oncology,[45] sepsis,[46] and primary care[47]). We will evaluate these models by comparing their performance when constructed using various process-mining algorithms. The performance of the models will be measured by:[48]

1. *Replay fitness*, a measure of how many traces from the log can be reproduced in the process model, with penalties for skips and insertions; range 0 – 1.
2. *Precision*, a measure of how 'lean' the model is at representing traces from the log. Lower values indicate superfluous structure in the model; range 0 – 1.
3. *Generalisation*, a measure of generalisability as indicated by the redundancy of nodes in the model. The more redundant the nodes, the more variety of possible traces that can be represented; range 0 – 1.

Patient flow as defined by the best-performing process model will be described using the multi-level approach of Kurniati *et al.*,[49] which include activity, trace and model measures. As all sub-studies progress, other measures of patient flow might be suggested for study, e.g. patient-level measures.

Sub-study 3: Patient safety

This sub-study will contribute to objectives 1 ('Impact Evaluation') and 2 ('Process Evaluation') by directly evaluating the differences in patient-safety outcomes before and after the implementation of the Command Centre. The evaluation will use longitudinal data-analysis methods, e.g. including interrupted time-series analysis and latent growth modelling. We will model trends behaviour before, during and after the implementation of the Command Centre, with consideration for the onset of the COVID-19 pandemic. Unobserved confounders will be handled by including a control site from the same geographical region that uses the same electronic health record system, but which does not use a Command Centre.

For peer review only

We will approach the analysis in a responsive manner, adding or removing interrupts in response to unfolding understanding of the Command Centre's implementation, based on our qualitative process evaluation. Candidate variables of interest will include the Patient Safety Indicators from the Agency for Healthcare and Research Quality, e.g. pressure ulcer rate, in-hospital fall with hip fracture rate, post-operative sepsis rate.[50] These will be supplemented by variables of interest informed by the qualitative sub-studies and early PPIE workshops. See **Table 1** for the potential list of variables for analysis. The final included set of variables will be defined based on availability of historic data, data quality and relationship with the intervention logic, as established through the parallel qualitative work.

We assume that patient safety is influenced by the flow of patients and the quality of information (as encoded in electronic health records). Therefore, we also intend to use the aforementioned sub-studies on data quality and patient flow to inform clinically meaningful outcomes logically related to patient safety. These outcomes will be subject to longitudinal analysis like the patient safety outcomes.

For peer review only

Table 1 List of proposed variables for analysis

Variable	Patient Flow	Patient Safety	Data quality
Ambulance handover times	x		
Ambulance diversion rates	x		
Time to be seen	x		
Time to treat stroke patients		x	
Waiting time benchmarks e.g. 4hr/18hr	x		
Left without being seen rates		x	
Diagnostic process time	x		
Time to discharge	x		
Time to admission	x		
In-hospital transfers	x		
Hospital-acquired infections		x	
Falls in hospital		x	
Pressure sores in hospital		x	
Mortality in hospital		x	
Early discharges	x		
Length of stay	x		
Post-operative sepsis rate		x	
Mortuary crowding	x		
COVID bed availability	x		
Intensive Care Unit bed usage	x		
Readmission rates for same condition (within 48-72hrs)		x	
Number of patients awaiting surgery (inpatients/at home)	x		
Cancelled operations (electives and non-electives)	x		
Marked 'hospital discharge'			x
Completeness			x
Correctness			x
Currency			x

Sub-study 4: Ethnography and qualitative interview

This study will contribute to objectives 1 ('Impact Evaluation') and 2 ('Process Evaluation'). Ethnographic enquiry has been selected to facilitate deep understanding of the technology in its broader social and organisational context, including human experience, engagement and interaction.[51,52] We aim to achieve a comprehensive description of how the Command Centre is integrated and embedded within the broader sociotechnical hospital system through observation of

enacted working practices, communication, decision-making, and operating culture. In this sense, we will not simply be relying upon the model for the system implementation as planned by programme leads, but will explore the differences between work as intended and work as done[19] and describe any unintended consequences and implementation barriers as they emerge. The ethnographic and qualitative interview sub-study is comprised of three main research activities, detailed below.

Unstructured observations

We will conduct unstructured observations in the Command Centre to gather information of staff interactions with data, and the ways in which this influences decision-making. Through research field notes and interviews, we will seek to understand the role of the Command Centre in coordinating care, from the perspective of staff in and around the Command Centre. Two researchers will undertake up to 36 hours of observation, completed in up to 4-hour windows that represent different times of day and days of the week. Observation periods will be pre-specified through arrangement with Command Centre leads. In addition, the researchers will record incidents of observer effects to allow analysis of whether participants' awareness of the researchers' presence changed over time.[53] We will also explore behaviour and meaning around specific events, drawing upon the Critical Incident Technique.[54]

We will review emerging hospital policies and guidance related to the Command Centre (e.g. meeting minutes and operational procedures) where practicable as an alternative to data collection involving staff. A sampling framework to guide collection of documents will be informed through earlier qualitative interviews with Command Centre leads and iterated during the research process. Documents that meet inclusion criteria will be recorded in a document inventory and a data extraction template will be created to obtain the necessary information from the documents. The extracted data will be analysed through an inductive process to capture key developments in design and functioning of the Command Centre.

Longitudinal stakeholder and process evaluation:

For peer review only

Our process evaluation framework[55] will systematically explore the experiences, beliefs, and expectations from multiple user perspectives in relation to operational planning and delineate the trajectories by which patient safety, operational and other intermediary outcomes are impacted by Command Centre processes.

We will use qualitative research interviews at multiple time points within the Command Centre programme. This will include building upon the ethnographic work to explore interactions with prior theory concerning how the Command Centre might work. The evaluation will address the factors that govern engagement with and use of this technology, using technology adoption theory.[56] The evaluation will also address the efficiency and effectiveness of processes for generating new intelligence for decision making and quality improvement, at the level of the hospital. A key output will be a logic model to describe the Command Centre as a complex, health-informatics intervention.

Sampling will be theoretically driven, based upon emerging insights from the structured observations, and will include Command Centre programme leads, key roles working in the centre, clinical leads in frontline areas interacting with the Command Centre, and organisational level stakeholders representing senior information systems, operational strategy, clinical governance, and financial interests. Up to 20 interviews will be undertaken at the study site focusing on two timepoints: one during the early phase of the project and the second towards the end of data collection. Representation of comparable roles will be sought at the control site, for comparative analysis of how the implicated functions are delivered in conventional operational planning processes.

Structured observations:

We will undertake structured observations to inform use-cases of operational planning, control and decision-making in priority areas, at the hospital level. Our approach to structured observation will draw upon engineering use-case methodology[57] to understand usability of the system in context. We will follow key information from the Command Centre's visual displays to decision and actions taken by key professional roles. This will involve shadowing various roles, such as bed managers, risk

For peer review only

management, quality assurance, clinical leads and others, as they act and make decisions based on Command Centre information. We will produce up to six use cases, based upon 60 hours of observation. We will utilise the use cases identified in structured observations as a probe to compare operational planning, control and decision-making in specific priority areas, with and without the support of a centralised Command Centre function, to enrich our understanding of how a Command Centre operates within a health service context.

Field notes and interview transcripts will be entered into NVivo (version 12.6) software to facilitate data management. Data analysis will comprise both inductive and deductive analyses, employing frameworks derived from prior theory and a comparative perspective across the two study sites.[58,59]

Relevant theoretical frameworks for sense making in our analysis will include models of situational awareness,[60] operational command and control, [61–63] sociotechnical evaluation, [64] High Reliability Organisations[7,62,65] and resilience in healthcare.[66,67] From the perspective of Situational Awareness theory, for example, we will seek to understand how the Command Centre enhances human perception of the environment and events within time and space, including projection of future states, and facilitates comprehension of meaning.[60]

Sub-study 5: Cross-industry consultation and CIO survey

The sub-study will contribute to objective 3 ('Cross-industry Perspectives'). We will undertake a cross-industry review as part of our work, comprising qualitative literature review and consultation with subject-matter experts in a range of safety-critical domains. Such an approach has been applied successfully in previous work, which sought to elicit and apply knowledge from high-risk industry to the development of incident reporting systems in healthcare.[68]

Data collection will involve scoping the literature in a range of domains for conceptual and empirical models of causal mechanisms for centralised command and control. We will additionally consult with up to 10 industry experts, including representatives of similar command centre programmes in other

For peer review only

health systems, accessed through UK health care human factors and other professional networks. This process will be supported by the project's Study Steering Group. The results will be synthesised to inform analysis and interpretation of our data.

We will also conduct a survey of the perceptions of NHS information personnel in acute care across England and Wales to understand variations in electronic data-facilitated command and control within hospitals beyond the two research sites. The survey instrument will be informed by earlier qualitative research work and literature review. We will capture views on current practices in data-supported operational planning and control, the costs-benefits of investment in centralised command and control "centres", information/data readiness, implementation barriers and perceptions of the need for further development in this area.

The survey will be disseminated to NHS information personnel through the University of Leeds Online Surveys network. Data will be transferred into SPSS software to facilitate data analysis.

Sampling and recruitment

For the qualitative work, we will recruit relevant staff at the Command Centre and control site in key roles. NHS staff working in and around the Command Centre will be asked to take part in ethnographic observations. Up to 40 NHS staff will be interviewed, 20 from each site. We will sample ≤ 10 cross-industry experts for interviews, and ≤ 40 NHS hospital information personnel in England and Wales will be asked to take part in the survey. Sampling will be theoretically informed in accordance with qualitative research practices to maximise variation in stakeholder perspectives. Potential participants will be identified through clinical leads and early observations.

For the quantitative work stream, we will use complete sampling of electronic health records within relevant periods. The duration of relevant periods will be informed by the initial case description and unstructured observations in the qualitative work, which will sensitise us to the information handled by the Command Centre.

Consent and data handling

For peer review only

Research participants will be told that they do not have to take part in the research if they wish and that they can withdraw up to the point that their data has been anonymised (< 2 weeks following research interviews; < 1 week following survey). The quantitative work will analyse routinely-collected healthcare records data. These data will have been de-identified and processed by the hospitals' data teams and accessed via Connected Yorkshire – an ethically approved regional integration of healthcare and other data available for research purposes.[69]

Patient and public involvement and engagement

Patient and public involvement and engagement is an integral part of our study design, delivery and dissemination. Figure 1 shows how the PPIE work stream is engaged throughout all phases of the project. Pre-study PPIE included a patient-and-public representative as co-applicant (NS), input on research design by the PPIE Research Fellow at the NIHR Yorkshire and Humber Patient Safety Translational Research Centre, and an informal survey of visitors to the hospital in which the Command Centre was implemented.

Early project PPIE activity includes workshops at the Command Centre and control site to engage PPIE representatives to give lay perspectives on care coordination in hospitals, to inform the development of interview questions for hospital staff, and to suggest measures of patient safety. Representatives have been recruited by advertisement through the patient groups both associated and not associated with the Bradford hospital site. Representatives will be reimbursed in accordance with the NIHR standards and the INVOLVE framework, e.g. monetary or voucher reimbursement for contribution to workshops and additional reimbursement remote participation. Our PPIE co-applicant will support qualitative data analysis through review and further development of emerging themes in the dataset. Toward the end of the project, a joint workshop will host PPIE representatives from both hospital sites to help interpret findings and to draft a PPIE communication plan. Our PPIE Lay Leader and project co-applicant will also co-develop case descriptions with the qualitative research team and will co-author all publications to provide a PPIE perspective. We will provide ongoing feedback as part of our engagement work with local trust stakeholders, including patient representatives

For peer review only

Ethics and dissemination

This protocol has been approved by the University of Leeds Engineering and Physical Sciences Research Ethics Committee (#MEEC 20-016) and the NHS Health Research Authority (IRAS No.: 285933). The protocol was developed by a multi-disciplinary team of co-applicants, including PPIE representatives, and by the NIHR as part of the NIHR 19/16 HSDR Digital Technologies to Improve Health and Care funding processes.

At the end of the study, we will provide a report detailing design feedback to improve the implementation of the AI Command Centre at the Bradford site and more generally for the system supplier and the digital technology industry. Our results will be communicated through peer-reviewed publications in international journals and conferences.

To contribute to objective 4 (‘Synthesis and Dissemination’), we will involve the research team, project co-applicants, the Study Steering Committee, stakeholders, and PPIE representatives consolidate research findings, PPIE perspectives and Study Steering Committee insight into outputs to appropriate audiences.

After the project has completed, data that is approved to do so will be offered to the University of Leeds Research Data Repository, in accordance with our Data Management Plan (Appendix 3).

Limitations

This proposed protocol was planned prior to the COVID-19 pandemic, which has caused substantial changes to the structures and processes used in healthcare systems. Our sub-studies on data quality, patient flow and patient safety are intended to provide quantification of the influence of the Command Centre implementation but will be limited in their capability to distinguish such contributions from those motivated by the response to COVID-19. Our mixed-method approach and involvement from our international Study Steering Group will help to define the context of this turbulent period and to describe the processes of change in the hospitals studied. Under the epistemic

For peer review only

constraints of our pre-COVID, funder-approved protocol, we will interpret our research through these contextual descriptions.

Acknowledgements

We thank the PPIE representatives for their contribution to the conception, development, ongoing implementation and future communication of this project. This research is supported by the National Institute for Health Research (NIHR) Yorkshire and Humber Patient Safety Translational Research Centre (NIHR Yorkshire and Humber PSTRC). The views expressed in this article are those of the authors and not necessarily those of the NHS, the NIHR, or the Department of Health and Social Care.

Authors' contributions

JB, CMC, CMI and OJ conceptualised the study. NS contributed a patient and public perspective for decisions about research design, acceptability, relevance, conduct and governance from study conception to dissemination plans. JB, CMC, CMI, NS, IH, TL, RR and OJ contributed to the design of the protocol, reviewed the initial funding bid and responded to funder queries. CMI and CMC prepared the first draft of the protocol manuscript and TM led revisions. All authors have been involved in revising the work for important intellectual content and have approved the final versions for publication.

Funding statement

This project is funded by the National Institute for Health Research Health Service and Delivery Research Programme (NIHR129483).

Competing interests

None declared.

For peer review only

References

1 Stange KC. The problem of fragmentation and the need for integrative solutions. *Ann Fam Med* 2009;**7**:100–3. doi:10.1370/afm.971

2 Castro-Sánchez E, Charani E, Drumright LN, *et al.* Fragmentation of care threatens patient safety in peripheral vascular catheter management in acute care- A qualitative study. *PLoS One* 2014;**9**. doi:10.1371/journal.pone.0086167

3 Nguyen L, Bellucci E, Nguyen LT. Electronic health records implementation: An evaluation of information system impact and contingency factors. *Int J Med Inform* 2014;**83**:779–96. doi:10.1016/j.ijmedinf.2014.06.011

4 Lau F, Bartle-Clar JA, Bliss G, *et al.* Improving usability, safety and patient outcomes with health information technology: From research to practice. In: Lau F, Bartle-Clar JA, Bliss G, *et al.*, eds. *Information Technology and Communications in Health, ITCH 2019*. Victoria, Canada: : IOS Press 2019. https://pdfs.semanticscholar.org/8141/c5c3f62e1346cc200fbaf429d988b7130e04.pdf#page=521

5 Sujan MA, Scott P, Cresswell K. Digital health and patient safety: Technology is not a magic wand. *Health Informatics J* Published Online First: 2019. doi:10.1177/1460458219876183

6 Ramanujam R, Rousseau DM. The challenges are organizational not just clinical. *J Organ Behav* 2006;**27**:811–27. doi:10.1002/job.411

7 LaPorte T, Consolini P. Working in Practice but Not in Theory: Theoretical Challenges of "High-Reliability Organizations. *J Public Adm Res Theory* 1991;**1**:19–48.

8 Roberts K, Madsen P, Desai V, *et al.* A case of the birth and death of a high reliability healthcare organisation. *Qual Saf Heal Care* 2005;**14**:216.

9 Holwell S, Checkland P. An information system won the war. *IEE Proc Softw* 1998;**145**:95–9. doi:10.1049/ip-sen:19982194

10 Espejo R. Complexity and change: Reflections upon the cybernetic intervention in Chile, 1970-1973. *Syst Pract* 1990;**3**:303–13. doi:10.1007/BF01062734

11 Buch VH, Ahmed I, Maruthappu M. Artificial intelligence in medicine: current trends and future possibilities. *Br J Gen Pract* 2018;**68**:143–4. doi:10.3399/bjgp18X695213

12 Johns Hopkins Medicine. The Johns Hopkins Hospital Launches Capacity Command Center to Enhance Hospital Operations. 2016.https://www.hopkinsmedicine.org/news/media/releases/the_johns_hopkins_hospital_launches_capacity_command_center_to_enhance_hospital_operations (accessed 26 Jan 2021).

13 Bell, John. Life sciences: industrial strategy. Published Online First: 2017.https://assets.publishing.service.gov.uk/government/uploads/system/uploads/attachment_data/file/650447/LifeSciencesIndustrialStrategy_acc2.pdf

14 UK AI COUNCIL. AI Roadmap. 2021.https://www.gov.uk/government/publications/ai-

For peer review only

roadmap

15 Sidek YH, Martins JT. Perceived critical success factors of electronic health record system implementation in a dental clinic context: An organisational management perspective. *Int J Med Inform* 2017;**107**:88–100. doi:10.1016/j.ijmedinf.2017.08.007

16 Black AD, Car J, Pagliari C, *et al.* The Impact of eHealth on the Quality and Safety of Health Care: A Systematic Overview. *PLoS Med* 2011;**8**:e1000387. doi:10.1371/journal.pmed.1000387

17 Johnson OA, Fraser HSF, Wyatt JC, *et al.* Electronic health records in the UK and USA. *Lancet* 2014;**384**:954. doi:10.1016/S0140-6736(14)61626-3

18 Protti D. Comparison of IT in general practice in 10 countries. *Heal Q* 2007;**10**:107–16.

19 Hollnagel E, Wears RL, Braithwaite J. From Safety-I to Safety-II: A White Paper. 2015.https://www.england.nhs.uk/signuptosafety/wp-content/uploads/sites/16/2015/10/safety-1-safety-2-whte-papr.pdf

20 Hollnagel E. *Safety-I and safety-II: the past and future of safety management*. Ashgate 2018.

21 Endsley MR. Measurement of situation awareness in dynamic systems. *Hum Factors* 1995;**37**:65–84. doi:10.1518/001872095779049499

22 Endsley MR. Situation awareness misconceptions and misunderstandings. *J Cogn Eng Decis Mak* 2015;**9**:4–32. doi:10.1177/1555343415572631

23 Weick KE, Sutcliffe K, Obstfeld D. Organizing for high reliability: Processes of collective mindfulness. In: Sutton R, Staw B, eds. *Research in Organizational Behavior*. JAI Press 1999. 81–123.

24 Cantu J, Tolk J, Fritts S, *et al.* High Reliability Organization (HRO) systematic literature review: Discovery of culture as a foundational hallmark. *J Contingencies Cris Manag* 2020;**28**:399–410. doi:10.1111/1468-5973.12293

25 Aboumatar HJ, Weaver SJ, Rees D, *et al.* Towards high-reliability organising in healthcare: A strategy for building organisational capacity. *BMJ Qual Saf* 2017;**26**:663–70. doi:10.1136/bmjqs-2016-006240

26 Sturmberg JP, O'Halloran DM, Martin CM. Understanding health system reform - A complex adaptive systems perspective. *J Eval Clin Pract* 2012;**18**:202–8. doi:10.1111/j.1365-2753.2011.01792.x

27 Sturmberg JP. Want improved quality? Improve your systems. *J Eval Clin Pract* 2020;**26**:1530–3. doi:10.1111/jep.13461

28 Stulberg JJ, Schäfer WLA, Shallcross ML, *et al.* Evaluating the implementation and effectiveness of a multi-component intervention to reduce post-surgical opioid prescribing: Study protocol of a mixed-methods design. *BMJ Open* 2019;**9**:1–11. doi:10.1136/bmjopen-2019-030404

29 Grove A, Clarke A, Currie G, *et al.* Advancing clinical leadership to improve the implementation of evidence-based practice in surgery: a longitudinal mixed-method study protocol. *Implement Sci* 2020;**15**:1–10. doi:10.1186/s13012-020-01063-2

30 Ha NHL, Chan I, Yap P, *et al.* Mixed-method evaluation of CARITAS: A hospital-To-community model of integrated care for dementia. *BMJ Open* 2020;**10**:1–12. doi:10.1136/bmjopen-2020-039017

For peer review only

31 Schmidt B, Watt K, McDermott R, *et al.* Assessing the link between implementation fidelity and health outcomes for a trial of intensive case management by community health workers: A mixed methods study protocol. *BMC Health Serv Res* 2017;**17**:1–6. doi:10.1186/s12913-017-2320-2

32 Mann R, Beresford B, Parker G, *et al.* Models of reablement evaluation (MoRE): A study protocol of a quasi-experimental mixed methods evaluation of reablement services in England Organization, structure and delivery of healthcare. *BMC Health Serv Res* 2016;**16**:1–9. doi:10.1186/s12913-016-1600-6

33 Manojlovich M, Adler-milstein J, Harrod M, *et al.* The Effect of Health Information Technology on Health Care Provider Communication: A Mixed-Method Protocol. *JMIR Res Protoc* 2015;**4**:e72. doi:10.2196/resprot.4463

34 Allan S, Mcleod H, Bradstreet S, *et al.* Understanding Implementation of a Digital Self-Monitoring Intervention for Relapse Prevention in Psychosis: Protocol for a Mixed Method Process Evaluation. *JMIR Res Protoc* 2019;**8**:e15634. doi:10.2196/15634

35 Sun Y, Luo R, Li Y, *et al.* App-Based Salt Reduction Intervention in School Children and Their Families (AppSalt) in China Protocol for a Mixed Methods Process. *JMIR Res Protoc* 2021;**10**:e19430. doi:10.2196/19430

36 Ward M, McAuliffe E, Wakai A, *et al.* Study protocol for evaluating the implementation and effectiveness of an emergency department longitudinal patient monitoring system using a mixed-methods approach. *BMC Health Serv Res* 2017;**17**:1–10. doi:10.1186/s12913-017-2014-9

37 Farre A, Cummins C. Understanding and evaluating the effects of implementing an electronic paediatric prescribing system on care provision and hospital work in paediatric hospital ward settings: A qualitatively driven mixed-method study protocol. *BMJ Open* 2016;**6**:1–9. doi:10.1136/bmjopen-2015-010444

38 Van Huizen LS, Dijkstra P, Halmos GB, *et al.* Does multidisciplinary videoconferencing between a head-and-neck cancer centre and its partner hospital add value to their patient care and decision-making? A mixed-method evaluation. *BMJ Open* 2019;**9**. doi:10.1136/bmjopen-2018-028609

39 Department of Health. Research governance framework for health and social care. *Health Soc Care Community* 2005;**10**:1–54.

40 Weiskopf NG, Bakken S, Hripcsak G, *et al.* A data quality assessment guideline for electronic health record data reuse. *J Electron Heal Data Methods* 2017;**5**:1–19.https://egems.academyhealth.org/articles/10.5334/egems.218/

41 Weiskopf NG, Bakken S, Hripcsak G, *et al.* Appendix B_A data quality assessment guideline for electronic health record data reuse. *J Electron Heal Data Methods* 2017;**5**:1–19. doi:10.13063

42 Fernandez-Llatas C, Munoz-Gama J, Martin N, *et al.* Process Mining in Healthcare. In: Fernandez-Llatas C, ed. *Interactive Process Mining in Healthcare. Health Informatics*. Champaign, IL: : Springer 2021. doi:10.1007/978-3-030-53993-1_4

43 Mans RS, van der Aalst WMP, Vanwersch RJB. *Process Mining in Healthcare: Evaluating and Exploiting Operational Healthcare Processes*. Springer 2015. doi:10.1007/978-3-319-16071-9

44 Mans RS, Reijers HA, Van Genuchten MJIM, *et al.* Mining Processes in Dentistry. In: *IHI'12: proceedings of the 2nd ACM SIGHIT: International Health Informatics Symposium*. New York: : ACM 2012. 379–88.

For peer review only

45 Baker K, Dunwoodie E, Jones RG, *et al.* Process mining routinely collected electronic health records to define real-life clinical pathways during chemotherapy. *Int J Med Inform* 2017;**103**:32–41. doi:10.1016/j.ijmedinf.2017.03.011

46 Mannhardt F, Blinde D. Analyzing the trajectories of patients with sepsis using process mining. In: *CEUR Workshop Proceedings*. 2017. 72–80.http://ceur-ws.org/Vol-1859/bpmds-08-paper.pdf

47 Williams R, Ashcroft DM, Brown B, *et al.* Process mining in primary care: Avoiding adverse events due to hazardous prescribing. *Stud Health Technol Inform* 2019;**264**:447–51. doi:10.3233/SHTI190261

48 Van der Aalst W, Adriansyah A, Van Dongen B. Replaying history on process models for conformance checking and performance analysis. *Wiley Interdiscip Rev Data Min Knowl Discov* 2012;**2**:182–92. doi:10.1002/widm.1045

49 Kurniati AP, McInerney CD, Zucker K, *et al.* A multi-level approach for identifying process change in cancer pathways. In: *Conference: 17th International Conference on Business Process Management (BPM 2019)*. Vienna, Austria: 2019.

50 McDonald KM, Romano PS, Geppert J, *et al. Measures of Patient Safety Based on Hospital Administrative Data - The Patient Safety Indicators*. Agency for Healthcare Research and Quality 2002.

51 Greenhalgh T, Swinglehurst D. Studying technology use as social practice: the untapped potential of ethnography. *BMC Med* 2001;:45.

52 Holloway I. *Qualitative research in health care*. Maidenhead: : OUP 2005.

53 McDonald S. Studying actions in context: a qualitative shadowing method for organizational research. *Qual Res* 2005;**5**:455–73.

54 Flanagan J. The Critical Incident Technique. *Psychol Bull* 1954.

55 Moore GF, Audrey S, Barker M, *et al.* Process evaluation of complex interventions: Medical Research Council guidance. *BMJ* 2015;**350**.

56 Greenhalgh T, Wherton J, Papoutsi C, *et al.* Beyond adoption: A new framework for theorizing and evaluating nonadoption, abandonment, and challenges to the scale-up, spread, and sustainability of health and care technologies. *J Med Internet Res* 2017;**19**. doi:10.2196/jmir.8775

57 Kulak D, Guiney E. *Use Cases: Requirements in Context*. Addison-Wesley 272 2012.

58 Randell R, Dowding D. Organisational influences on nurses' use of clinical decision support systems. *Int J Med Inform* 2010;**79**:412–21. doi:10.1016/j.ijmedinf.2010.02.003

59 Ritchie J, Spencer L. Qualitative data analysis for applied policy research. In: Bryman A, Burgess E, eds. *Analysing qualitative data*. Oxon: : Routledge 1994.

60 Endsley MR. Toward a Theory of Situation Awareness in Dynamic Systems. *Hum Factors* 1995;**37**:32–64.

61 Faraj S, Xiao Y. Coordination in Fast-Response Organizations. *Manage Sci* 2006;**52**:1155–69.

62 Weick KE, Sutcliffe KM. *Managing the Unexpected: Assuring High Performance in an Age of Uncertainty*. 2nd ed. San Francisco, CA: : Wiley 2001.

63 Jordan S, Johannessen I. Mindfulness and Organizational Defenses: Exploring Organizational

For peer review only

and Institutional Challenges to Mindfulness. In: Ngnoumen A, Langer E, eds. *The Wiley Blackwell Handbook of Mindfulness*. John Wiley & Sons 2014. 424–42.

64 Holden RJ, Karsh B-T. The Technology Acceptance Model: Its past and its future in health care. *J Biomed Inform* 2010;**43**:159–72.

65 Roberts KH, Madsen P, Desai V, *et al.* A case of the birth and death of a high reliability healthcare organisation. *Qual Saf Heal Care* 2005;**14**:216–20. doi:10.1136/qshc.2003.009589

66 Benn J, Healey AN, Hollnagel E. Improving performance reliability in surgical systems. *Cogn Technol Work* 2008;**10**:323–33. doi:10.1007/s10111-007-0092-x

67 Hollnagel E. Resilience engineering and the systemic view of safety at work: Why work-as-done is not the same as work-as-imagined. *Bericht zum 58 Kongress der Gesellschaft für Arbeitswiss vom* 2012;**22**:19–24.

68 Benn J, Koutantji M, Wallace L, *et al.* Feedback from incident reporting: Information and action to improve patient safety. *Qual Saf Heal Care* 2009;**18**:11–21.

69 Research BI for H. Connected Yorkshire. 2021.https://www.bradfordresearch.nhs.uk/our-research-teams/connected-bradford/

Figure legend:

Figure 1: Schematic overview of the research project's components. BRI = Bradford Royal Infirmary, CIO = Chief Information Officer, HS&DR = National Institute for Health Research (NIHR) Health Service and Deliver Research funding programme, PPIE = Patient and Public Involvement and Engagement, WS = work stream

For peer review only

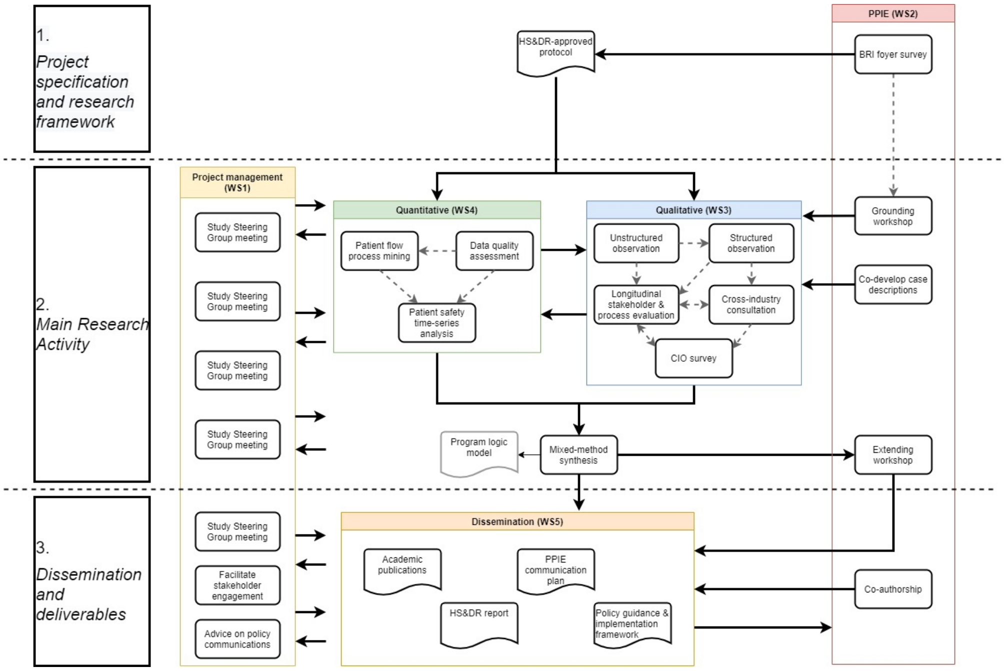

Figure 1: Schematic overview of the research project's components. BRI = Bradford Royal Infirmary, CIO = Chief Information Officer, HS&DR = National Institute for Health Research (NIHR) Health Service and Deliver Research funding programme, PPIE = Patient and Public Involvement and Engagement, WS = work stream

460x304mm (72 x 72 DPI)

Appendix 1-Research Aim-Activities Matrix

Below is a matrix that indicates which research activities address which research aims and objectives

				Patient-safety time-series analysis	Patient-flow process mining	Data quality assessment	Unstructured observation	Structured observation	Stakeholder interview	Cross-industry consultation	CIO survey	Mixed-method synthesis	Dissemination
**Aim 1**	Evaluate the impact of the CC on patient safety, hospital operational efficiency and related organisational processes.	**a**	Describe (qualitatively) and evaluate (statistically) any effect on patient safety, including monitoring of deteriorating patients and sub-optimal care pathways, risk of harm due to cancellation/delays and situational awareness in safety-critical areas such as the emergency department.	X			X	X	X				
		**b**	Describe (qualitatively) and evaluate (statistically) any effect on patient flow, including capacity-demand ratio, transfer delays, bed utilisation, timely discharge and cancellations of scheduled care.		X		X	X	X				
		**c**	Qualitatively investigate any effect on organisational processes, such as situational awareness, operational decision-making, risk and coordination/communication across organisational units, from multiple stakeholder perspectives.				X	X	X				
**Aim 2**	Understand the process of implementation and integration of the CC and associated data infrastructure and organisational processes within the primary study site	**a**	Using qualitative methods, describe the process of development and implementation of the CC, including critical implementation factors and any unintended consequences.				X	X	X				
		**b**	Through ethnographic methods, investigate the process by which the CC system and outputs are embedded at all levels of the organisation, from frontline operations to strategic quality and safety governance.					X					
		**c**	Develop and validate a logic model for this health informatics intervention that maps system preconditions, processes, technology and outcomes, at the primary study site.									X	
		**d**	Describe (statistically and qualitatively) the effect of the CC implementation on the local data environment, including data infrastructure, quality and integration (i.e. system interoperability).			X	X	X	X				
**Aim 3**	Elicit cross-sector and cross-industry perspectives on hospital command and control technologies to contextualise the findings from the	**a**	Review and understand command and control processes in non-healthcare safety-critical operations and the key principles and contextual factors that may influence transferability of these models into a hospital setting.							X			

iew only

	primary study site for broader application.	b	Survey the perceptions of senior health informatics professionals on current command and control processes, viability of novel “mission-control” systems, data readiness and potential implementation barriers.								X		
**Aim 4**	Synthesise the research findings into practical outputs that will engage service stakeholders and inform future investment and practice	a	Share learning concerning cross-industry and empirical findings on the costs-benefit of investment within NHS management and Chief Information Officer networks.										X
		b	Construct an empirically-informed implementation framework that describes contextual factors and implementation pathway for development of centralised, data-driven mission-control systems in acute care, including data infrastructure maturity.									X	

For peer review only

Appendix 2- Study Steering Group membership

Below are the details of the members of the Study Steering Committee for the NIHR HS&DR project entitled "Evaluating the safety and patient impacts of an AI Command Centre in the NHS" (NIHR129483).

Name	Affiliation	Reason for nomination
Iain Buchan (Chair)	University of Liverpool; University of Manchester (Honorary)	Executive Dean, Institute of Population Health Sciences; Director of Digital Strategy and Partnerships, Liverpool Health Partners; Chair of Public Health and Clinical Informatics
Paul Charnley	Wirral NHS Teaching Hospitals Trust	Director of IT and Information at Wirral NHS Teaching Hospitals Trust
Sarah Culkin	NHS England; NHSX	Artificial Intelligence lead for NHS England
Cindy Fedell	Regional Chief Information Officer at Northwestern Ontario Hospitals	Former Chief Information Officer at Bradford Teaching Hospitals NHS Foundation Trust
Hamish Fraser	Brown University, USA	A health informatics academic and clinical lead for OpenMRS, the most-widely used open-source medical record system
Charles Koo	Envive Technology, Ltd.; Visiting Scholar (Stanford University); Visiting Expert (National Taiwan University)	Implemented large scale Artificial-Intelligence, hospital systems at six hospitals in Shanghai, China.
Farah Magrabi	Macquarie University	Associate Professor Australian Institute of Health Innovation
Sue Mason	Sheffield University; Sheffield NHS Teaching Hospitals Trust	Professor of Emergency Medicine at Sheffield University and clinician at Sheffield NHS Teaching Hospitals Trust
Mark Sujan	University of Warwick; Human Factors Everywhere	Human Factors expert
Hilary Thompson	NIHR Yorkshire and Humber Patient Safety Translational Research Centre	Patient representative and NIHR YH PSTRC Lay Leader
Sean White	NHS Digital	Senior Safety Engineer
Patrick Waterson	Loughborough University	Reader in Human Factors and Complex Systems

For peer review only

University of Leeds Data Management Plan (DMP) Template

Researcher Name	Owen Johnson
Project Title	Evaluating the safety and patient impact of an Artificial Intelligence Command Centre in the UK National Health Service
Faculty	Engineering and Physical Sciences
KRISTAL Reference Number (if applicable)	118684
Supervisor(s) name (if applicable)	n/a
Funder	National Institute for Health Research
Scheme	
Research Start Date	1st March 2021
Research End Date	30th Sept 2022
Ethical review number	Not available, yet
DMP review due	3rd May 2021

Date	Version	Author	Change notes
11th Nov 2020	V1.0	Ciarán McInerney	n/a
16th Dec 2020	V2.0	Ciarán McInerney and Carolyn McCrorie	Added data management plan for qualitative work.
10th Feb 2021	V3.0	Ciarán McInerney and Carolyn McCrorie	Added details of the standards with which Yorkshire and Humber Care Record's Google Cloud Platform are compliant.

Please provide a brief overview of your project including proposed research methods

AI Command Centre in Bradford NHS Bradford Teaching Hospitals NHS Foundation Trust is implementing an artificial intelligence (AI) command centre in Bradford Royal Infirmary, which is regarded as the first of its kind in Europe. The command centre follows an approach successfully used in the USA (Johns Hopkins, Baltimore) and Canada (Humber River, Toronto) to provide real-time, rapid response to clinical, management and patient-flow challenges. Similar to an air traffic control command centre, hospital staff work together in a purpose-built operations room and monitor a 'wall of analytics' of high-definition screens that display real-time data from the hospital's clinical systems. The team review, monitor and react to the 'big picture' of how efficiently patients are flowing through the hospital, where bottlenecks might occur, where pressure is building and where safety breaches are predicted. The command centre software makes use of AI technologies that are refined through operation to provide increasingly more intelligent alerts and warnings. There is a very limited evidence base for this form of digital technology in hospitals but an increasing belief that AI can and should play a key role in transforming and modernising the NHS. Our research team in the NIHR Yorkshire & Humber Patient Safety Translational Research Centre (YH-PSTRC) are based in Bradford and in a unique position to collect and study the evidence. Research Framework We propose a mixed-method study with three phases over 18 months, commencing in 2020. In the first phase, we will carry out initial scoping work with relevant stakeholders. The second and third phases will involve longitudinal case studies describing the impact of an AI command centre on safety. We will also

For peer review only - http://bmjopen.bmj.com/site/about/guidelines.xhtml

conduct a mixed-method comparison with hospitals such as Calderdale and Huddersfield NHS foundation Trust, which has shared systems and learning through close collaboration with Bradford. Outputs Our aim in this study is to provide a robust academic evaluation of the AI Command Centre so that other hospitals and healthcare providers can consider how best to exploit this emerging technology. The findings from our study will be reported to the NHS trusts, including local dissemination of findings. They will also be reported in a white paper for distribution to NHS Chief Information Officers, in academic publications, to NIHR as part of the annual report for funding body for the YH-PSTRC and as funders for this specific project and dissemination of findings via the NIHR PSTRCs' networks.

1. What data will be produced? What data will be used from other sources?

With respect to the quantitative component of the project:

1. Study on patient safety.
Collect:

- **What?** - Routinely-collected electronic health record data that informs the measures of patient safety identified during early qualitative work. This data is created and stored in Bradford Teaching Hospital NHS Trust's electronic health record system, Cerner Millennium. This data will be de-identified and aggregated within the Yorkshire Health and Care Record, from which we will request extracts.
- **Why?** - This data is created by the hospital system being studied and so reflects the safety performance of the hospital system.

Create:

- **What?** - Summary statistics of the distribution and dynamics of patient-safety measures at both sites. Visualisations of the values. Models of variable dynamics. We will also create R scripts, which are text documents containing instructions to run commands in the R statistical programme.
- **Why?** - The measures of patient-safety are the variables of interest in this study. The R scripts are needed to process the data. The visualisations will facilitate investigations and some will be used to communicate the research.

2. Study on patient flow.
Collect:

- **What?** - Routinely-collected electronic health record data created and stored in Bradford Teaching Hospital NHS Trust's electronic health record system, Cerner Millennium. This data will be de-identified within the Yorkshire Health and Care Record, from which we will request extracts.
- **Why?** - Pseudonymised patient identifier, a description of a clinical event, and the timestamp for the event are the minimal requirements to build a process model of a patient flow through a hospital.

Create:

For peer review only - http://bmjopen.bmj.com/site/about/guidelines.xhtml

- **What?** - We will create R scripts, which are text documents containing instructions to run commands in the R statistical programme. These R scripts will produce R files containing process models and summary statistics describing the process models. Visualisations of the models and values.
- **Why?** - The models describe patient flow, which is a marker of patient safety. These models need to be evaluated so we compute summary statistics of their performance. The R scripts are needed to process the data. The visualisations will facilitate investigations and some will be used to communicate the research.

3. Study on data quality.
Collect:

- **What?** - Routinely-collected electronic health record data that are presented or used to inform variables that are presented on the AI Command Centre tiles. This data is created and stored in Bradford Teaching Hospital NHS Trust's electronic health record system, Cerner Millennium. Identifying the variables of interest will require an audit of the tiles of interest, which will be conducted in early qualitative work.
- **Why?** - This data is created by the hospital system being studied. We wish to assess how the quality of this data might have changed as the Command Centre was gradually implemented.

Create:

- **What?** - We will be using the Weiskopf et al. (2017) tool for assessing the quality of the data, which will create the data indicated in the table below, as per their 3x3 data-quality matrix. Each cell of the matrix will inform a visualisation of the data. We will also create R scripts, which are text documents containing instructions to run commands in the R statistical programme.

	Complete	*Correct*	*Current*
Patients	Counts and percentages of Variables, Times with recorded data, and Overall points of data present.	Details of expected value limits for the variables across patients. A citation for the source of the expectation. Counts and percentages of patients whose data are within the value limits.	Details of the expected period within which the data were recorded. A citation for the source of the expectation. For each non-static variable, Counts and percentages of patients whose data do not fall within the desired date range. Summary statistics of the discrepancies between actual recording dates and desired date ranges (mean, median, mode, 1st quartile, 3rd quartile, interquartile range, minimum,

For peer review only - http://bmjopen.bmj.com/site/about/guidelines.xhtml

			maximum, standard deviation, variance)
Variables	Counts and percentages of Patients with the variable, Times with the variable present, and Overall points of data present.	Details of criteria that indicate concordance for each variable. A citation for the source of the expectation. Counts and percentages of patients who meet each criterion. Counts and percentages of patients whose data violate any of the criteria.	Details of the expected sequence or intervals between events. A citation for the source of the expectation. Counts and percentages of patients that meet expectations, for each variable. Counts and percentages of patients that meet all expectations.
Times	Counts and percentages of Patients with data recorded for that time, Variables with data recorded for that time, and Overall points of data present.	Details of criteria that indicate valid changes over time, for each variable. A citation for the source of the expectation. Counts and percentages of patients who meet each criterion. Counts and percentages of patients whose data violate any of the criteria.	The I-score for each variable for which regularity is to be assessed. Summary statistics for the I-score across all variables (mean, median, mode, 1st quartile, 3rd quartile, interquartile range, minimum, maximum, standard deviation, variance). The IxN-score to measure "effective data points".

- **Why?** - The data created using the Weiskopf et al. (2017) tool for assessing the quality are the variables of interest in this study. The R scripts are needed to process the data. The visualisations will facilitate investigations and some will be used to communicate the research.

With respect to the qualitative component of the project:

1. Case description and unstructured observation

Collect:

- **What?** -Ethnographic observations within the Command Centre (CC) Unit. Up to 36 hours over 4-hour periods.
- **Why?** -In order to immerse and sensitise the research team to the context of hospital operational command and control. The data will help us to understand events and actions as they unfold from the actor's perspective (and the meanings that CC users attach to them).

Create:

- **What?** -Researcher field notes

For peer review only

CC BY NC SA

- **Why?** -To draw upon the concepts of Grounded Theory in which we will adopt an inductive qualitative analysis approach to understand way in which the CC system integrates within the broader hospital information and operational planning systems in a formal model grounded in our data.

2. System-wide structured observation

Collect:

- **What?** -Ethnographic observations within and beyond the Command Centre. Up to 10 hours observation each of specific tracer issues/professional roles that represent interaction with CC processes and outputs.
- **Why?** -In order to explore the impact of the CC beyond the operations room and at all levels of the organisation, including micro-level (frontline clinical workflow in specific specialties), meso-level operational planning (e.g. bed management) and macro-level strategic planning (e.g. use of data in quality and safety governance).

Create:

- **What?** -Researcher field notes
- **Why?** -To draw upon concepts of Realist Evaluation in our analysis to understand usability of the system in context.

3. Longitudinal stakeholder and process evaluation

Collect:

- **What?** -Qualitative research interviews with up to 24 staff relative to the initiative
- **Why?** -To evaluate the efficacy of the system from multiple user perspectives

Create:

- **What?** -Interview transcripts
- **Why?** -To draw upon a process evaluation framework approach to analysis in order to understand intervention mechanisms, implementation processes, interaction with context and overall outcomes.

4. Cross-industry study

Collect:

- **What?** -Qualitative research interviews with up to 10 consultants in safety critical industries
- **Why?** -To elicit and apply knowledge from high-risk industry to the development of strategies for implementing command and control centres to improve quality and safety

Create:

- **What?** -Interview transcripts

For peer review only - http://bmjopen.bmj.com/site/about/guidelines.xhtml

- **Why?** -To draw upon a process evaluation framework approach to analysis in order to understand intervention mechanisms, implementation processes, interaction with context and overall outcomes.

5. Survey study

Collect:

- **What?** -Survey of a sample of Chief Information Officers in acute care across England and Wales
- **Why?** -To capture views on current practices in data-supported operational planning

Create:

- **What?** -Survey responses captured through UoL Online Surveys
- **Why?** -The data is required to understand variations in electronic data-facilitated command and control beyond the 2 research sites

2. Where will data be stored? How will data be structured? Include file formats and approximate volume.

Where will data be stored?

The electronic health records used in the quantitative work will not be copied for storage or back-up, by the research team. Instead, it will be hosted by the Yorkshire and Humber Care Record and accessed via their virtual research environment. The Yorkshire and Humber Care Record system is built on Google Cloud technology with Identity Access Management following the principle of least privilege, i.e. minimum permissions of access and functionality. Google Cloud technology is compliant with GDPR, ISO/IEC 27001, ISO/IEC 27017, ISO/IEC 27018, ISO/IEC 27701, NHS Digital Commercial Third-Party Information Governance Requirements, UK's Cloud Security Principles. Further details are available at https://cloud.google.com/security/compliance.

Toward the end of the project, summative research output for publications and all R scripts used for data processing will be exported from the Yorkshire and Humber Care Record portal and stored on the University of Leeds SAN (Storage Area Network), which comprises enterprise level disk storage and file servers located in physically secure data centres with appropriate fire suppression equipment. Snapshots are taken every day at 10pm (and accessible for 1 month). A second level of snapshots is taken every month and are kept for 11 months. Snapshots are user recoverable from the desktop.

A full back-up to tape is taken once every month and an incremental copy to backup tape is taken every night (and kept for 28 days). Every quarter, the most recent set of full dump tapes are moved to a long-term storage facility where they are kept for 12 months. Tapes are initially stored in on-campus fireproof safes and then moved to off-campus secure locations. The SAN is located behind the University's Institutional firewall to protect against external attacks.

During the life of the qualitative work, the data will be stored on the University of Leeds SAN. The audio-recording equipment will be encrypted. Survey data will be stored in UoL Online Surveys

After the project has completed, data will be offered to the University of Leeds Research Data Repository (Research Data Leeds) or another appropriate data repository service in order to ensure the data can be shared, reused and cited beyond the end of the project. Research Data Leeds holds deposited data for a minimum of 10 years and datasets are associated with digital object identifiers (DOIs).

How will data be structured?

cc BY NC SA

For peer review only - http://bmjopen.bmj.com/site/about/guidelines.xhtml

With respect to the ‘collected’ quantitative data (in so far as 'data collection' refers to the method by which data is obtained):

- **Format** - The data is expected to be accessible in the Yorkshire Health and Care Record portal as a CSV file or in one of the SQL database file types, which we would subsequently transform into a CSV file type.
- **Volume** - The size of the dataset will be informed mostly by the count of patients, rather than by the count of variables. Both hospital sites under study typically see in the range of 70,000 - 80,000 unique patients every month, each with at least two events (admission and discharge). This translates to a volume of data in the order of millions of observations across multiple variables. File sizes are likely to be in the order of MBs.

With respect to the created quantitative data:

- **Format** - The data will be stored as R files and CSV files, outputted using RStudio. The R format will facilitate analysis while the CSV formats are preferred to transferability to other software. All visualisations will be stored in PNG and JPEG formats. The JPEG formats are have smaller file sizes and will be used only when the higher quality PNG format is not supported.
- **Volume** - Likely no more than low double figures of megabytes.

With respect to the collected qualitative data:

- **Format** – The interview transcripts and field notes will be stored as files within NVivo 12 version. Survey data will be stored in SPSS software.
- **Volume** – The size of the data set will be informed by the number of interviews undertaken, size of the field notes and number of responses to the survey.

With respect to the created qualitative data:

- **Format** - The interview transcripts and field notes will be stored as files within NVivo 12 version. Survey data will be stored in SPSS software.
- **Volume** – The size of the data set will be informed by the number of interviews undertaken, size of the field notes and number of responses to the survey.

3. Access to data during the project. Give details of collaborators and any controls.

During the life of the quantitative work, access to the 'collected' data will be controlled by the Yorkshire and Humber Care Record, with whom our research team will have a contract stipulating the terms of use of the Yorkshire and Humber Care Record portal. The created data that will occasionally be exported and stored on University of Leeds' Storage Area Network. These data will only be accessible by university staff with user privileges and password access.
During the life of the qualitative work, the data will be stored on UOL SAN. Only members of the research team will have access via user privileges and password access.
After the project has completed, the data that will be stored with the data repository service will only be accessible on request and following approval criteria that will be co-developed by the research team and the data repository service.

4. Ethics and legal compliance: are there any ‘special’ requirements for your data? Any contractual or consent issues? Key policies (internal and external)

For peer review only - http://bmjopen.bmj.com/site/about/guidelines.xhtml

Consent – all interviewees will give informed consent for the interview to be audio-recorded and transcribed.

5. How will data be documented and described? Methodologies and protocols.

All folders will contain a README in a TXT file format that explains what files are in the folder. Each file will be named, its provenance (including source and steps taken to process it), and details of any restrictions on sharing. No formal standard will be adhered to.

6. Training and support

All researchers have completed UoL training in Information Security Essential.

7. What are the plans for data sharing beyond project partners? Include justification if some of your data needs to be restricted. Include data and code. Include repository.

After the project has completed, the data that will be stored with a data repository service will only be accessible on request and following approval criteria that will be co-developed by the research team and the data repository service. Data will be made available via the University of Leeds data repository and, where possible, as supplementary material accompanying academic publications.

The electronic health records used in the quantitative work will not be made available outside of the Yorkshire and Humber Care Record in which it was accessed.

8. What Intellectual Property will be generated? How will IP be protected and exploited?

We are not expecting to generate Intellectual Property beyond the academic outputs produce vai the research process.

9. Who is responsible for managing the data? What resources will you need?

The Principal Investigator will have ultimate responsibility for data management during the project but the quantitative and qualitative study leads will have day-to-day responsibilities. After the project, the data will be managed by the University of Leeds data repository staff.

10. Ongoing data curation / data housekeeping - you may find it useful to include a retention table

All summative data used to communicate research findings in published output will be stored in the University of Leeds data repository. This repository holds deposited data for a minimum of 10 years and datasets are associated with digital object identifiers (DOIs).

End of Project

At the end of a project and/or before you leave the institution, you should ensure that data and research materials are deposited with the School or a trusted data repository and documented in such a way that they can be found and understood.

Dataset name	Location	Person responsible

Adapted from University of Cambridge DataTrain project & University of Edinburgh MANTRA project

CC BY NC SA

Research Data Leeds http://researchdata.leeds.ac.uk/ email: researchdataenquiries@leeds.ac.uk
Oct 2019

University of Leeds Data Management Plan (DMP) Template: Prompt Sheet

1. What data will be produced or used? (Including original software)

- ☐ What physical data will you study? (e.g. artefacts, samples, paper archives, etc.)
- ☐ What digital data will you generate? (e.g. field-notes, images, spreadsheets, audio interviews, survey data, annotated bibliography, etc.)
- ☐ What original software will you generate?
- ☐ What third party data will you reuse?

2. Where will data be stored? How will data be structured?

- ☐ Estimate how much data you will produce over time – do you have enough storage?
- ☐ Do you know what University storage is available and how to access it?
- ☐ What file formats and software will you use?
- ☐ Do you have a logical file naming convention and directory structure?
- ☐ How will you use versioning so you can identify the current version of documents / data?
- ☐ How will data generated in the field be saved to safe University storage?

3. Access to data during the project. Give details of collaborators and any controls.

- ☐ Have you discussed data sharing with your research collaborators/ supervisor?
- ☐ Who needs to access data during the research? How will they access data?
- ☐ Do you need a data sharing agreement? (see also section 4.)

4. Ethics and legal compliance: are there any 'special' requirements for your data?

- ☐ Have you read the University's Information Protection Policy? Data must be assessed for sensitivity and storage in line with this policy https://it.leeds.ac.uk/it?id=kb_article&sysparm_article=KB0011140
- ☐ Are you familiar with the University's advice on data protection and GDPR? https://dataprotection.leeds.ac.uk/
- ☐ Does your research funder have specific data management and sharing requirements?
- ☐ Are there other policies and protocols you need to be aware of and observe? For example, NHS codes of practice?
- ☐ Will you anonymise your data?
- ☐ Should some data be destroyed? When and how?
- ☐ How and where will you record any participant consents and/or contractual requirements which impact data management and sharing? The DMP can be a good place to record this information.

5. How will data be documented and described? Methodologies and protocols.

- ☐ Will others understand your data? Write documentation. Make sure table and spreadsheet values are clearly labelled.
- ☐ What information about data collection methodology will be recorded?
- ☐ Is it important for the research to be reproducible? Why/why not? What additional documentation will be required?
- ☐ Will you write software? Where will this be documented and stored for future use?

Adapted from University of Cambridge DataTrain project & University of Edinburgh MANTRA project
CC BY NC SA

Research Data Leeds http://researchdata.leeds.ac.uk/ email: researchdataenquiries@leeds.ac.uk
Oct 2019

6. Training and support

- ☐ What training do you need for data gathering, organisation, analysis or presentation?
- ☐ Are there relevant courses available at the University? Online? Who can provide support?

7. What are the plans for data sharing beyond project partners?

- ☐ Have you considered reasons for and against sharing data? Will data be openly available to everyone or will there be access restrictions?
- ☐ If your research involves people, have you obtained appropriate consent for data sharing?
- ☐ Can your data be released immediately, or should you embargo (delay access to) the data?
- ☐ How long will / should data be available for?
- ☐ Will you use a data repository? Which one? Are there subject specific data repositories in your field?

8. What IPR will be generated? How will IPR be protected and exploited?

- ☐ Will you be applying for a patent? Will your research have commercial applications? Do you need to contact the Commercialisation team in the Research and Innovation Service?
- ☐ Have you read the University Intellectual Property Policy? http://ris.leeds.ac.uk/downloads/download/600/university_of_leeds_ipr_policy

9. Who is responsible for managing the data? What resources will you need?

- ☐ Who is responsible for data at different stages in its lifecycle?
- ☐ On projects with complex data management requirements, different types of role should be specified.
- ☐ How will best practice and guidance be shared across the project partners?
- ☐ Are sufficient resources (skills, people, storage, technology) available to deliver your plan?

10. Ongoing data curation / data housekeeping - you may find it useful to include a retention table

- ☐ What data will you keep? Who decides?
- ☐ Where will data be kept and for how long.
- ☐ Who needs to know what data exists on the network, where it is, how it should be managed and how long it should be retained?

For peer review only

Don't forget to review and update your data management plan regularly

---

But I don't have any data! *Anything can become research data if it is used for research purposes – data is not just numbers on a spreadsheet. Think creatively about the materials you are using and producing: what could be shared with other researchers who are interested in your work; what could be reused to produce new insights? Any evidence or material which underpins or sheds light on your findings, your academic publications, your thesis or your project can be considered research data.*

Adapted from University of Cambridge DataTrain project & University of Edinburgh MANTRA project

cc BY NC SA
